# Clinicopathological data and the role of miRNA expression in patients with pheochromocytomas/paragangliomas

**DOI:** 10.3389/fendo.2025.1716806

**Published:** 2025-12-04

**Authors:** Foteini Thanasoula, Maria P. Yavropoulou, Georgios Kyriakopoulos, Athanasios Papatheodorou, Chrysostomos Maltezos, Konstantinos Maltezos, Vasiliki Vasileiou, Eva Kassi, Gregory Kaltsas, Anna Angelousi

**Affiliations:** 1 First Department of Internal Medicine, European Reference Network on Rare Endocrine Conditions (ENDO-ERN), Laikon General Hospital, Medical School, National and Kapodistrian University of Athens, Athens, Greece; 2 Endocrinology Department and Diabetological Center, Alexandra General Hospital, Athens, Greece; 3 Department of Propaedeutic and Internal Medicine, European Reference Network on Rare Endocrine Conditions (ENDO-ERN), Laikon General Hospital, National and Kapodistrian University of Athens, Athens, Greece; 4 Department of Pathology, Evangelismos Hospital, Athens, Greece; 5 Department of Medical Research, 251 Hellenic Air Force & VA General Hospital, Athens, Greece; 6 Vascular Surgery Department, General Hospital of Athens, “K.A.T”, Athens, Greece; 7 Department of Biological Chemistry, Medical School, National and Kapodistrian University of Athens, Athens, Greece

**Keywords:** pheochromocytoma, paraganglioma, miRNA expression, biomarkers, metastatic disease, PDL1 expression, MSI (microsatellite instability), tissue

## Abstract

**Background:**

Pheochromocytomas and paragangliomas (PPGLs) are rare neuroendocrine tumors with variable behavior and metastatic potential. Reliable prognostic biomarkers are needed to improve risk stratification and long-term management. Emerging data suggest a role for microRNAs (miRNAs) and immune checkpoint pathways (or inhibitors) in tumor aggressiveness.

**Objective:**

To evaluate the expression of five candidate miRNAs (miR-15a, miR-16, miR-101, miR-183, and miR-483-5p) and programmed death ligand-1 (PD-L1) in PPGL tissues, and assess their associations with clinicopathological features, genetic status and outcomes.

**Methods:**

A retrospective cohort of 130 patients with PPGLs was analyzed, including 53 PPGL tumor and 20 normal adrenal medulla tissues (controls). MiRNA expression was assessed by RT-qPCR in 53 formalin-fixed paraffin-embedded samples (FFPE). PD-L1 and microsatellite instability (MSI) were evaluated by immunohistochemistry in FFPE samples. Associations with tumor type and size, functionality, Ki-67 index, grading scores (PASS, GAPP), metastatic status, localization, and genotype were examined.

**Results:**

Overall, 21.5% (28/130) of patients developed metastases during a median period of follow-up of 45 months and 35 out of the 61 tested (57.4%), harbored pathogenic germline mutations. In tumor samples (n=53), PD-L1 expression was observed in 18.9% (10/53) whereas no MSI expression was detected. MiR-483-5p was the most consistently upregulated marker in biochemically negative and in high–Ki-67 tumors along with significant up regulation in metastatic PPGL, supporting its role in cellular proliferation and metastatic potential. MiR-183 and miR-101 were overexpressed in pheochromocytomas (PHEOs) with high PASS and Ki-67 indices, while miR-15a and miR-16 displayed higher levels in non-metastatic tumors.

**Conclusion:**

MiR-483-5p emerges as a promising biomarker of aggressive PPGL behavior, while other miRNAs reflect distinct biological behaviors. PD-L1 expression in a subset of cases highlights immune checkpoint inhibition as a potential therapeutic strategy. Prospective validation is warranted.

## 1 Introduction

Pheochromocytomas (PHEOs) are rare neuroendocrine tumors arising from chromaffin cells of the adrenal medulla, with an estimated incidence up to 0.9 cases per 100,000 individuals annually (1). According to the 2004 World Health Organization (WHO) classification, tumors originating from extra-adrenal sympathetic or parasympathetic paraganglia are designated as paragangliomas (PGLs) (2). Collectively, these entities are referred to as pheochromocytomas/paragangliomas (PPGLs), with approximately 80% located in the adrenal medulla and 20% in extra-adrenal sites. An increasing proportion of PPGLs are discovered incidentally during imaging performed for unrelated indications, accounting for 5% of adrenal incidentalomas (3). The diagnosis of PPGLs remains challenging, particularly for nonsecretory or clinically “silent” tumors that often go undetected. Head and neck (HNPGLs) are secretory in only 3–4% of cases compared with 67–80% for abdominal PPGLs (4). In previous decades, over 50% of PPGLs were diagnosed at autopsy, whereas this rate has now fallen to less than 10% due to advances in functional imaging (5).

Approximately 40% of PPGLs are associated with hereditary tumor syndromes, including familial paraganglioma syndromes (SDHx mutations), von Hippel-Lindau disease (VHL), multiple endocrine neoplasia type 2 (RET), and neurofibromatosis type 1 (NF1), among others. To date, more than 12 genetic syndromes and 22 PPGL driver genes have been identified, and up to 50% of metastatic cases harbor germline mutations (6).

The prediction of metastatic potential remains a major clinical challenge, as no reliable histopathological features can distinguish tumors with aggressive behavior. Scoring systems such as the Pheochromocytoma of the Adrenal Gland Scaled Score (PASS) and the Grading System for Adrenal Pheochromocytoma and Paraganglioma (GAPP) demonstrate strong negative predictive values (99% and 96%, respectively) for tumors with low-risk scores, but have limited positive predictive values for high-risk scores tumors (7). Reflecting the potential for all PPGLs to metastasize, the 2017 WHO classification abandoned the terms “benign” and “malignant,” recommending instead the designation “metastatic” for tumors with spread to non-chromaffin sites, including bone, lymph nodes, liver, and lung (8).

The incidence of metastatic disease varies by tumor site and genotype, affecting approximately 10% of PHEOs and 15–35% of abdominal PGLs, with higher rates observed in SDHB mutation carriers. Metastases may be synchronous (35%) or, more commonly, metachronous (65%), developing at a median of 5.5 years after diagnosis, with rare cases occurring decades later (9). Prognosis remains poor, with a reported 5-year mortality of 43–63% (10), underscoring the need for lifelong surveillance and improved therapeutic strategies (11, 12).

In this context, the identification of biomarkers predictive of aggressive disease is of paramount importance. Recent studies have implicated several microRNAs (miRNAs), notably miR-15a, miR-16, miR-101, miR-183, and miR-483-5p, in tumor aggressiveness and metastatic potential (6, 13, 14). Additionally, immune-regulated mechanisms, particularly those involving programmed death ligands (PD-L1, PD-L2) influenced by tumor hypoxia, may contribute to disease progression (15).

The current study presents the clinicopathological data in a cohort of 130 PPGLs patients treated at the Centre of Excellence for Adrenal Tumors, in Laikon General Hospital, investigating the expression of selected miRNAs and the PD-L1/CTLA4Abs in 53 paraffin-embedded PPGLs tumors from a total of 48 patients.

## 2 Materials and methods

### 2.1 Participants

A total of 130 patients with histologically confirmed PPGLs were enrolled in the study from September 2022 to December 2024. Epidemiological, clinical and biochemical data as well as histopathological parameters were retrospectively recorded from the patients’ medical records. Patients without a histological report of PPGL were excluded from the study. All relevant data are presented in **Supplementary Table 1**. This project received approval from the Internal Ethical Review Committee of the Laiko General Hospital (protocol number 489, 18/07/2022). All patients/controls provided written informed consent prior to inclusion. All procedures were in accordance with the Declaration of Helsinki (1964) and its later amendments.

### 2.2 Tissue samples

**Figure 1:**
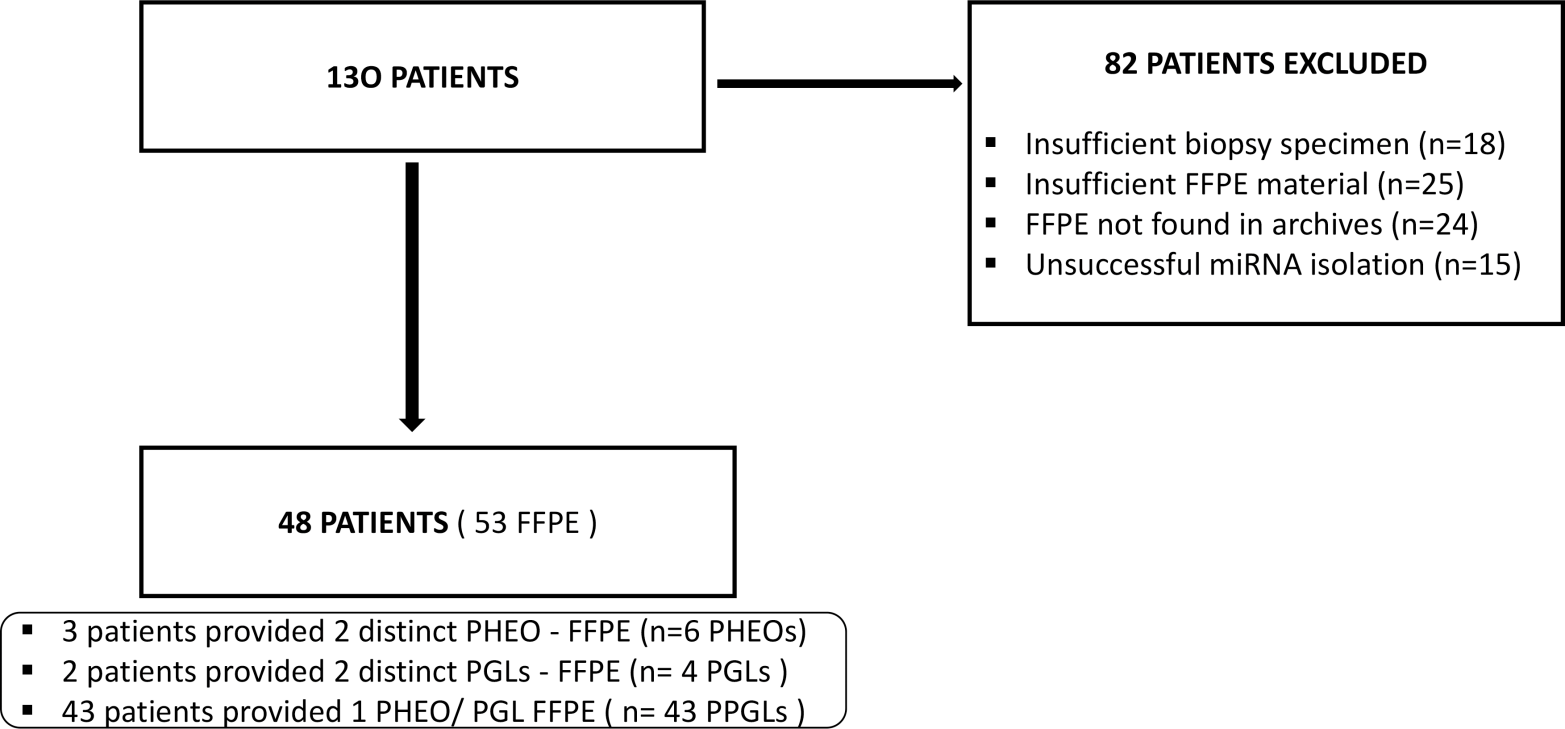
Flow chart illustrating the inclusion and exclusion process resulting in the final 48 PPGL patients from the initial 130 cases in the study. FFPE, Formalin-Fixed Paraffin-Embedded; PGLs, Paragangliomas; PHEOs, Pheochromocytomas; PPGLs; Pheochromocytomas and Paragangliomas.

Of the 130 patients included in the study, tumor tissue was ultimately available for analysis in 48 cases. Thirty- eight patients were referred from centers across the country to our department for a second opinion. Tissue samples were requested from all cases. However, in 18 cases only bioptical specimen was available and thus was not sufficient for further analysis similarly, in a further 25 cases, although they had fully resected tumor, FFPE specimens had insufficient material for further analysis. In 24 patients FFPE were not found in our archives (returned to patients), whereas in the remaining 15 patients miRNA isolation was not successful. Consequently, 53 FFPE tissue samples from 48 PPGL patients were analyzed, as shown in **Figure 1**. Among these, tissues from two distinct PHEOs were available from three patients, and from two distinct PGLs in two patients. The control group consisted of 20 individuals (5 men and 15 women-1:3) with a similar median age of 52 years (range, 28–80 years). All controls had histologically confirmed intact adrenal medulla and underwent adrenal or renal surgery for adrenocortical adenoma (n = 16), renal cell carcinoma (n = 2), angiomyolipoma (n = 1), and adrenal ganglioneuroma (n = 1).

#### 2.2.1 miRNA expression analysis

We evaluated the expression level of a panel of microRNAs-miRNA 483-5p, miRNA 183, miRNA 101, miRNA 15a, and miRNA 16-in a total of 53 available FFPE PPGL tumors and 20 normal adrenal medulla FFPE tissues (controls). Five 10 μM thick sections from each sample were used for purification of total RNA, including miRNA, from FFPE tissue sections using an QIAGEN miRNeasy FFPE kit (Qiagen, Hilden, Germany), according to the manufacturer’s instructions. After the elution, the quantity of miRNA was evaluated with a Qubit™ 4 Fluorometer (Invitrogen by Thermo Fisher Scientific, USA) using a Qubit™ microRNA Assay Kit. Using 20ng of miRNAs in a 20 μL reaction, the Qiagen miRCURY LNA RT kit was utilized for cDNA synthesis. One microliter of UniSp6 RNA Spike-In template was introduced to each reaction for the RNA isolation and cDNA synthesis quality control of the miRCURY LNA miRNA PCR assays. The reverse transcripts were diluted in a 1:60 ratio with RNase-free water, and the analysis was carried out with Qiagen miRCURY LNA miRNA PCR Assays, according to the manufacturer’s instruction (Qiagen, Hilden, Germany), on the Rotor-Gene Q 5plex HRM Platform with the Rotor-Gene Q Series Software Version: 2.3.5.

The expression was calculated according to the 2^-ΔΔCT^ method (16).

#### 2.2.2 Histopathological and immunohistochemical analysis

All histopathological and immunohistochemical analyses of FFPE tissues regarding microsatellite instability (MSI) and PDL1 were performed in the same laboratory (Evaggelismos Hospital) and assessed by the same pathologist either in the context of initial diagnosis or as a second opinion.

Immunohistochemical analysis for CgA and Syn was performed in all FFPE tissues as per protocol, using anti-CgA antibody (DAKO, clone DAK-A3) and anti-Synaptophysin antibody (DAKO, clone DAK-Synap).

##### 2.2.2.1 Immunohistochemistry for PD-L1

PD-L1 expression was evaluated using the PD-L1 IHC 22C3 pharmDx antibody (DAKO). From each FFPE block, six 4-μm serial sections were prepared: two for PD-L1 and four for MSI analysis. Adequate neoplastic cellularity was confirmed on hematoxylin–eosin staining. Positive controls included weak to strong staining of neoplastic or inflammatory cells (lymphocytes, macrophages), while absence of staining served as negative controls.

Sections were mounted on positively charged slides, deparaffinized at 60 °C for two hours, and rehydrated through graded alcohols. Antigen retrieval was performed with Dako EnVision™ FLEX Target Retrieval Solution (high pH) in a PT Link instrument (97 °C, 20 min). Slides were processed on the Dako Autostainer Link platform using the EnVision™ FLEX detection system. Endogenous peroxidase activity was blocked, followed by incubation with the primary antibody (PD-L1 or MLH1, PMS2, MSH2, MSH6) for 30 minutes. Detection employed a dextran polymer conjugated with secondary antibodies and peroxidase, visualized with 3,3′-diaminobenzidine (DAB). Counterstaining with hematoxylin was followed by dehydration, xylene clearing, and mounting.

##### 2.2.2.2 Scoring of PD-L1 expression

Staining intensity was graded as: 0: negative; 1+: weak membranous (complete or incomplete), 2+: moderate membranous staining,3+: strong membranous staining. Cytoplasmic staining of inflammatory cells was also considered positive. The Combined Positive Score (CPS) was calculated as the number of PD-L1–positive tumor and inflammatory cells divided by the total number of viable tumor cells, multiplied by 100 (17).

##### 2.2.2.3 Microsatellite instability analysis

MSI was assessed by nuclear expression of MLH1, PMS2, MSH2, and MSH6. Retained expression indicated microsatellite stability, whereas complete loss of either MLH1/PMS2 or MSH2/MSH6 was interpreted as MSI-high (MSI-H) (18).

##### 2.2.2.4 Tumor stratification

PPGLs were stratified using the Pheochromocytoma of the Adrenal Gland Scaled Score (PASS) (19) for PHEOs and the Grading of Adrenal Pheochromocytoma and Paraganglioma (GAPP) system (20) for both PHEOs and sympathetic PGLs.

### 2.3 Genetic analysis

Genetic testing via the Next-Generation Sequencing (NGS) method was requested for all patients with PPGLs, especially those with metastatic tumors.

### 2.4 Statistical analysis

The statistical analysis was performed using GraphPad Prism version 7.00 (GraphPad Software, La Jolla, CA, USA). In the descriptive analysis, categorical variables were expressed as their absolute and relative frequency. Numerical variables were presented as mean ± SD (Standard Deviation) for normally distributed data, otherwise by median and range. T-test or Mann-Whitney U test was used to compare the continuous variables between two groups, based on the results of Shapiro-Wilk normality test. For the comparison of numerical variables among multiple groups, one-way ANOVA or Kruskal -Wallis test followed by *post-hoc* analysis were used, as appropriate. The chi-square test or Fisher’s exact test was applied to compare categorical variables. Correlations between quantitative variables were investigated using either Pearson’s (r) or Spearman correlation coefficient (r_s_). P-values below 0.05 were considered statistically significant. Adjusted p-values for multiple comparisons were obtained using Dunn’s *post-hoc* test.

## 3 Results

### 3.1 Epidemiological, clinical and histopathological characteristics of PPGL patients

A total of 130 patients diagnosed with PPGLs were included in the study (**Supplementary Table 1**). The cohort comprised 54/130 males (41.5%) and 76/130 females (58.5%). Seventy-six out of the 130 patients (58.5%) were diagnosed with PGLs, 49/130 (37.7%) with unilateral PHEO, 4/130 (3%) with bilateral PHEOs and one patient (0.8%) was diagnosed with both PGL and PHEO. The median age at diagnosis was 48 years (range:15 to 78 years). Approximately half of the patients (53.8%) were symptomatic at presentation. Biochemical testing was performed in 102 of the 130 patients (78.5%), revealing that 59/102 (57.8%) had biochemically active tumors; 62.7% (37/59) of those were PHEOs and 37.3% (22/59) PGLs.

The median size of the primary tumor was 5.4 cm (range, min-max: 1–15 cm). The median proliferative Ki-67 index value was 3% (range, min-max: 1–20%). The median PASS for PHEOs was 6 (range, min-max: 1–12), while the median GAPP for both PHEOs and sympathetic PGLs score was 4 (range, min-max: 2–8).

Surgical resection was the primary therapeutic approach for the primary tumor in patients with PPGL, particularly in the absence of metastatic disease, and was performed in 95 out of 130 patients (73%), including 54.7% of those with PHEOs and 45.3% with PGLs. The median follow-up period of the study was 45 months, during which the overall survival rate was 96.1% (125/130 patients).

Metastatic disease was identified in 28 patients (21.5%); 6 patients (21.4%) had synchronous metastases at diagnosis, while 22 (78.6%) developed new metastases during the follow-up period (**Supplementary Table 2**).

In the management of metastatic disease, both local and systemic therapeutic approaches were employed. Local therapies included radiotherapy (25%) and repeated surgical resections (second surgery in 35.7%, third surgery in 10.7%). Systemic treatments such as chemotherapy, including Cyclophosphamide-Vincristine- Dacarbazine (CVD) or temozolomide, were used in 10 patients, in 7 of them as first-line treatment, radionuclide therapies (PRRTs or 131I-MIBG) were applied in 10 patients and molecular targeted therapies (MTT), including sunitinib and everolimus, were used in 5 patients. Data regarding the progression-free survival (PFS) according to the different treatments as well as data about the genetic analysis of the overall cohort are included in the **Supplementary Table 2**.

Genetic testing through NGS in blood samples (germline mutations) was conducted in 61 out of the 130 patients (46.9%), with disease-related mutations detected in 35 individuals (57.4%). Genetic analysis results are described in **Supplementary Table 3**.

### 3.2 Subcohort analysis in PPGLs FFPE tissues

#### 3.2.1 Epidemiological, clinical and genetic characteristics of the 48 PPGL patients

A total of 48 patients diagnosed with PPGLs were included in the study (**Table 1**). The cohort consisted of 17 males (35.4%) and 31 females (64.6%), with a median age at diagnosis of 50 years (range, 17–77 years). In total, 53 FFPE tissue samples were analyzed from these 48 patients. Among them, tissues from two distinct PHEOs were available for three patients, and from two distinct PGLs for two patients, resulting in 24 PHEO (24/53) and 29 PGL (29/53) tissue specimens overall.

**Table 1:** Epidemiological, clinical and histopathological characteristics of the 48 PPGL patients (n =53 FFPE).

Epidemiological clinical and genetic characteristics	Value
Median age (range: min-max), years Sex, male: female n (%)	50 (17–77) 17/48 (35.4%): 31/48 (64.6%)
Tumor type, (based on FFPE samples, n=53) - Patients with PHEOs, n=21 (18 with one, 3 with two) - Patients with PGLs, n=27 (25 with one, 2 with two)	24/53 (45.3%) 29/53 (54.7%)
Symptomatic at diagnosis, n (%)	20/48 (41.7%)
Biochemically secreting tumors, n (%)	20/48 (41.7%)
— PHEOs: PGLs among functional cases	15:5
- Metanephrines secreting	1/20 (5%)
- Normetanephrines-secreting	5/20 (25%)
- Both Metanephrines/Normetanephrines-secreting	14/20 (70%)
- Biochemically negative	28/48 (58.3%)
Metastatic disease, n (%)	10/48 (20.8%)
— Synchronous metastases	1 (10%)
— New metastases during follow-up	9 (90%)
Genetic testing performed, n (%)	21 (43.8%)
— Mutation-positive patients	10 (47.6%)
Median follow-up, months (range: min-max)	60 (12–228)
Survival rate	43/48 (89.6%)
Tissue characteristics	Value
Median tumor size, cm (range: min-max)	6.1 (1.2–14.2)
Ki-67 index %, median (range: min-max)	3 (1–13)
PASS score (PHEOs), median (range: min-max)	6 (1–12)
GAPP score (PHEOs-sympathetic PGLs), median (range: min-max)	4 (2-8)
MSI expression (MLH1, PMS2, MSH2, MSH6), n (%)	0/53 (0%)
PDL-1 expression (clone 22C3), n (%)	10/53 (18.9%)
CgA (+)	53/53 (100%)
Synaptophysin (+)	53/53 (100%)

FFPE, Formalin-Fixed Paraffin-Embedded; PHEOs, Pheochromocytoma; PGLs, Paragangliomas; PASS, Pheochromocytoma of the Adrenal Gland Scaled Score; GAPP, Grading of Adrenal Pheochromocytoma and Paraganglioma; PD-L1, programmed death ligand-1; MSI, microsatellite instability; CgA, Chromogranin A

At presentation, 20/48 patients (41.7%) were symptomatic. Biochemical evaluation demonstrated hormonally active tumors in 20/48 patients (41.7%), comprising 15 PHEOs and 5 PGLs.

Metastatic disease was documented in 10 out of 48 patients (20.8%). In all of them, except one, new metastases developed during follow-up. Genetic analysis was performed in 21/48 patients (43.8%); five cases harbored mutations of the *SDHX* family genes, 3 had *RET* mutations, and one had *NF1*, and *EPAS1* mutations, respectively. During a median follow-up of 60 months, the overall survival rate was 89.6%. The characteristics of the subcohort group of patients are presented in the **Table 1**.

#### 3.2.2 Tissue characteristics and immunohistochemical features

The median tumor size across the subcohort of 48 PPGLs patients was 6.1 cm (range, min-max: 1.2–14.2 cm). The proliferation index, measured by the Ki-67 labeling index, showed a median of 3% (range, min-max: 1–13%). Tumor grading scores revealed a median PASS of 6 (range, min-max: 1–12) among PHEO patients and a median GAPP score of 4 (range, min-max: 2–8) among PPGLs. Immunohistochemical staining for chromogranin A (CgA) and synaptophysin was performed in all tumor tissue samples, and both markers were positive in all cases. Further immunohistochemical analysis revealed no evidence of MSI in any of the 53 tumors evaluated for mismatch repair protein expression (MLH1, PMS2, MSH2, MSH6). PDL-1 expression (clone 22C3) was identified in 10 out of 53 (18.9%) FFPE tissues tested. Among the 10 tissues with positive PDL-1 expression, 5 were PHEOs and 5 HNPGL. One of these patients with positive PDL-1 expression had metastatic disease. Data are included in **Table 1**.

### 3.3 miRNA expression

#### 3.3.1 Analysis of miRNA expression in PPGLs tissues compared to controls

miR-483-5p demonstrated statistically significant overexpression in PPGLs compared to normal adrenal medulla (p < 0.0001). miR-15a (p = 0.113), miR-16 (p = 0.657), miR-101 (p = 0.184), and miR-183 (p = 0.965), did not show any statistical significance between PPGLs patients and normal adrenal medulla.

Comparison between PHEO tumors and controls demonstrated that miR-483-5p exhibited statistically significant upregulation in PHEO tumors compared to controls (p = 0.001). Additionally, miR-183 also showed borderline higher expression levels in PHEOs (p = 0.058). No significant differences were found for miR-15a (p = 0.259), miR-16 (p = 0.474) or miR-101 (p = 0.413).

Similarly, comparison between PGLs and controls revealed significantly overexpression of miR-483-5p (p = 0.0009) in PGLs, while no significant differences were found for miR-15a (p = 0.083), miR-16 (p = 0.872), miR-101 (p=0.248) and miR-183 (p = 0.369). A comparative analysis of miRNA expression between head and neck paragangliomas (HNPGLs) and non-HNPGLs revealed that miR-15a (p = 0.046) was significantly overexpressed in HNPGLs compared to non-HNPGLs whereas miR-16 was overexpressed in non-HNPGLs (p = 0.033). No significant differences were observed for miR-483-5p (p = 0.403), miR-101 (p = 0.324), and miR-183 (p = 0.411) between the two groups.

#### 3.3.2 miRNA expression and prognostic markers

**Figure 2:**
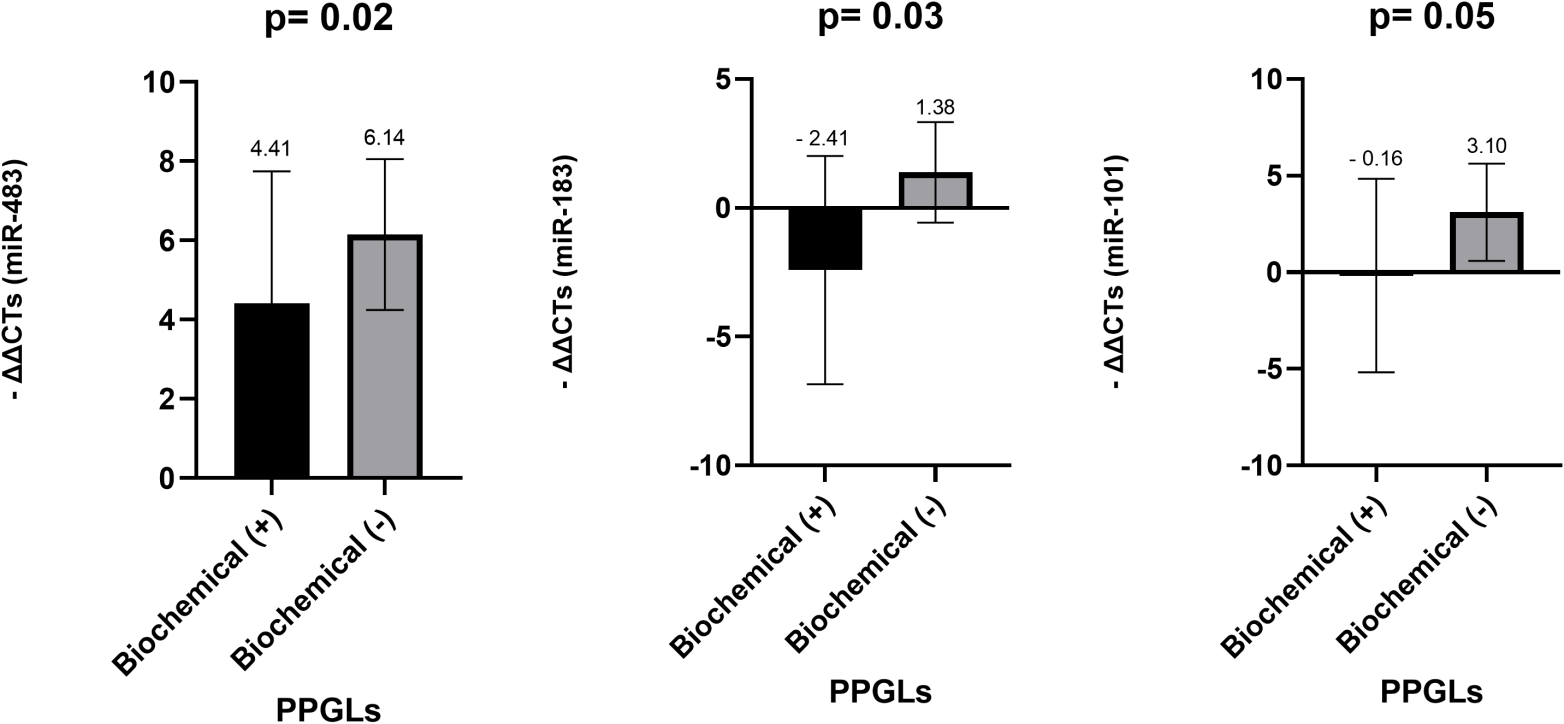
Comparison of miR-483-5p, miR-183 and miR-101 levels in FFPE tissues samples among biochemical positive and biochemical negative PPGLs. *Mann-Whitney test was applied to assess statistical differences between the groups. Error bars represent the standard deviation, and mean values are shown to illustrate the magnitude of each comparison. PPGLs, Pheochromocytomas and Paragangliomas; FFPE; Formalin-Fixed Paraffin-Embedded; Biochemical (+): tumors with plasma or urinary catecholamine/metanephrine/normetanephrine levels ≥ 3–5× upper normal limits (LC–MS/MS), Biochemical (-): tumors with normal plasma or urinary catecholamine/metanephrine/normetanephrine levels (LC–MS/MS).

miR-483-5p (p = 0.029) and miR-183 (p = 0.039) showed statistically significant overexpression in biochemical negative PPGLs compared to biochemical positive ones; miR-101 appeared to be marginally overexpressed in biochemical negative PPGLs with a borderline p-value (p = 0.052) (**Figure 2**). In contrast, miR-15a (p = 0.259) and miR-16 (p = 0.254) did not demonstrate any statistically significant differences between the two groups.

**Figure 3:**
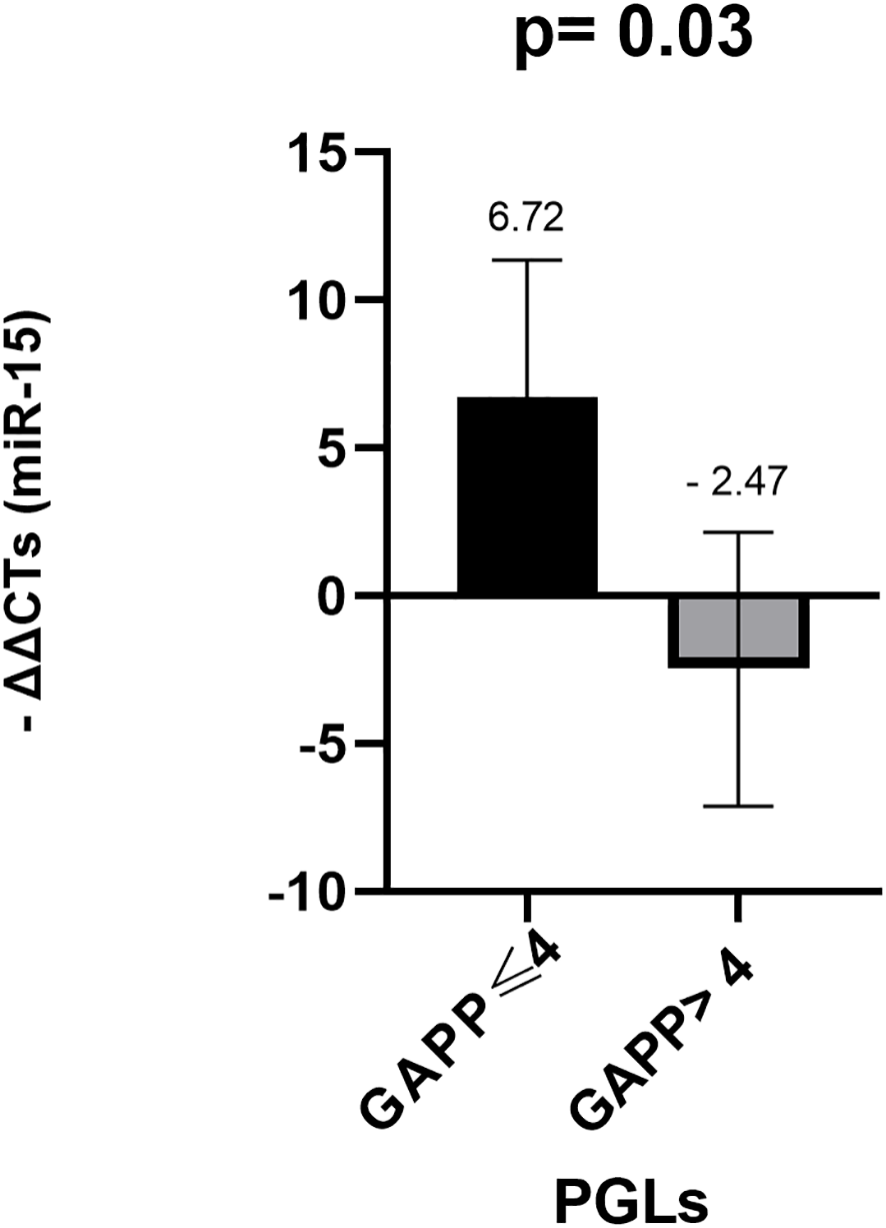
Comparison of miR-15a levels in FFPE tumors samples among PGLs with GAPP ≤ 4 and GAPP>4. *Mann- Whitney test was applied to assess statistical differences between the groups. Error bars represent the standard deviation, and mean values are shown to illustrate the magnitude of each comparison. FFPE, Formalin-Fixed Paraffin-Embedded; PGLs, Paragangliomas; GAPP, Grading system for Adrenal PHEO and PGL.

Regarding PGLs tumors, when stratified by GAPP score (≤4 vs. >4), only miR-15a exhibited a statistically significant difference between the two groups. Specifically, miR-15a (p = 0.031) was downregulated in PGLs with a GAPP score >4 (**Figure 3**).

**Figure 4:**
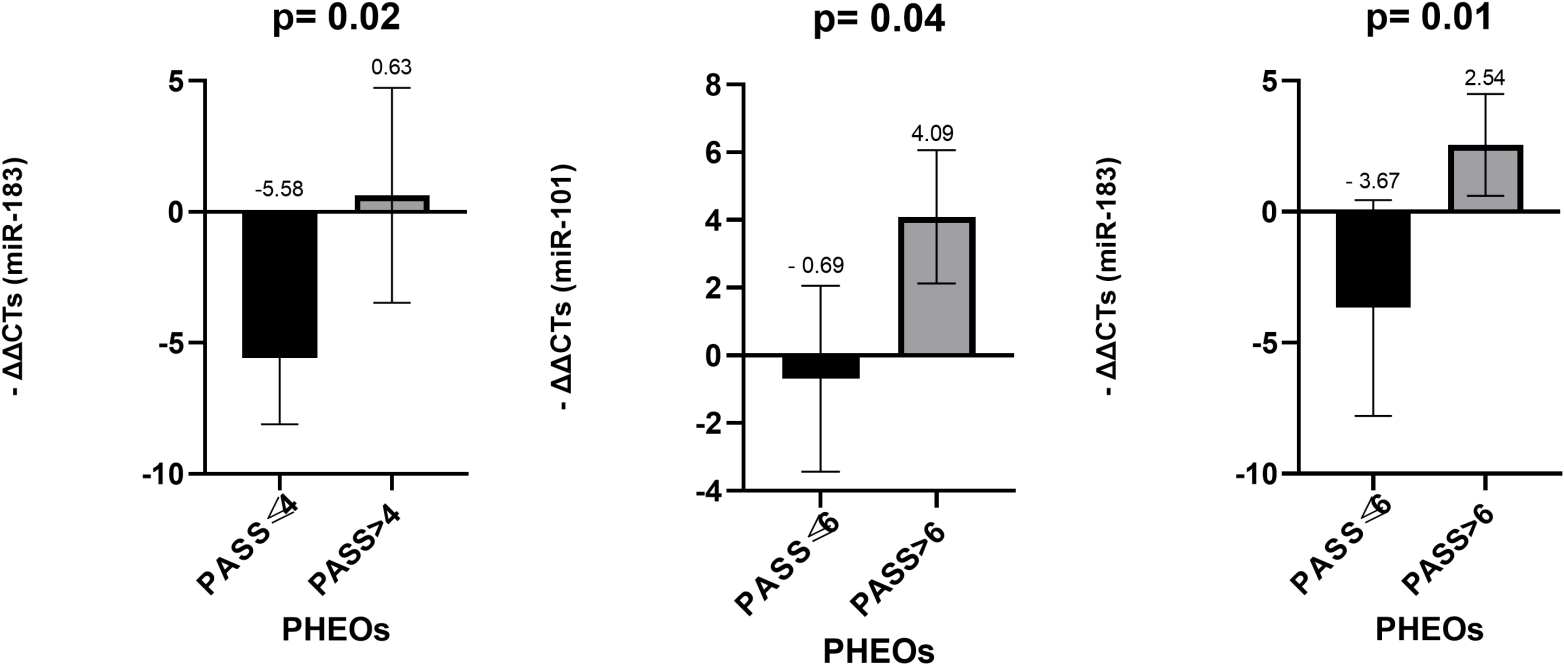
Comparison of miR-101 levels in FFPE among PHEOs with PASS ≤ 4 and PASS>4 and of miR-101 and miR-183 among those with PASS ≤ 6 and PASS>6. *Mann-Whitney test was applied to assess statistical differences between the groups. Error bars represent the standard deviation, and mean values are shown to illustrate the magnitude of each comparison. FFPE, Formalin-Fixed Paraffin-Embedded; PHEOs, Pheochromocytomas; PASS, Pheochromocytoma of the Adrenal gland Scaled Score.

Similarly, for PHEOs tumors, when stratified based on PASS (≤4 vs.>4), among the five miRNAs evaluated, only miR-183 was significantly overexpressed in tumors with PASS >4 (p = 0.028). Using a higher threshold (PASS ≤6 vs. >6), miR-101 (p = 0.048) and miR-183 (p = 0.015) were significantly overexpressed in PHEOs tumors with PASS >6 (**Figure 4**).

**Figure 5:**
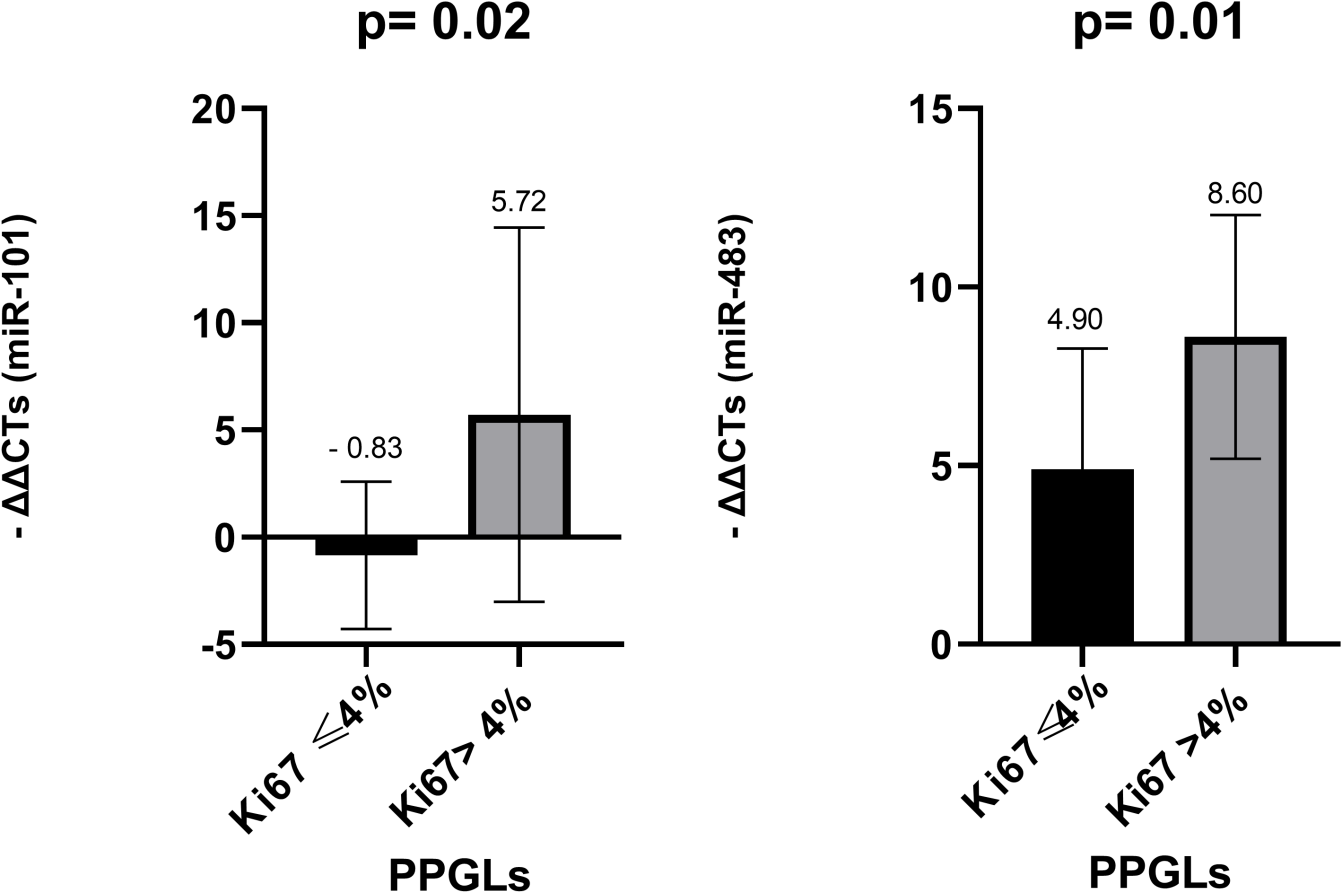
Comparison of miR-101 and miR-483-5p levels in FFPE among PPGLs with Ki67 index ≤ 4% and Ki67 index >4%. *Mann-Whitney test was applied to assess statistical differences between the groups. Error bars represent the standard deviation, and mean values are shown to illustrate the magnitude of each comparison. FFPE, Formalin-Fixed Paraffin-Embedded; PPGLs, Pheochromocytomas and Paragangliomas.

To evaluate whether tumor proliferation Ki-67 labeling index is associated with miRNA expression, patients were stratified into two groups those with Ki-67 ≤4% vs. >4%. No statistically significant differences were observed for miR-15a, miR-16, or miR-183 between the two groups (p = 0.518, 0.813 and 0.391, respectively). Both miR-483-5p (p = 0.016) and miR-101 (p = 0.021) showed statistically significant overexpression in tumors with Ki-67 >4% (**Figure 5**).

**Figure 6:**
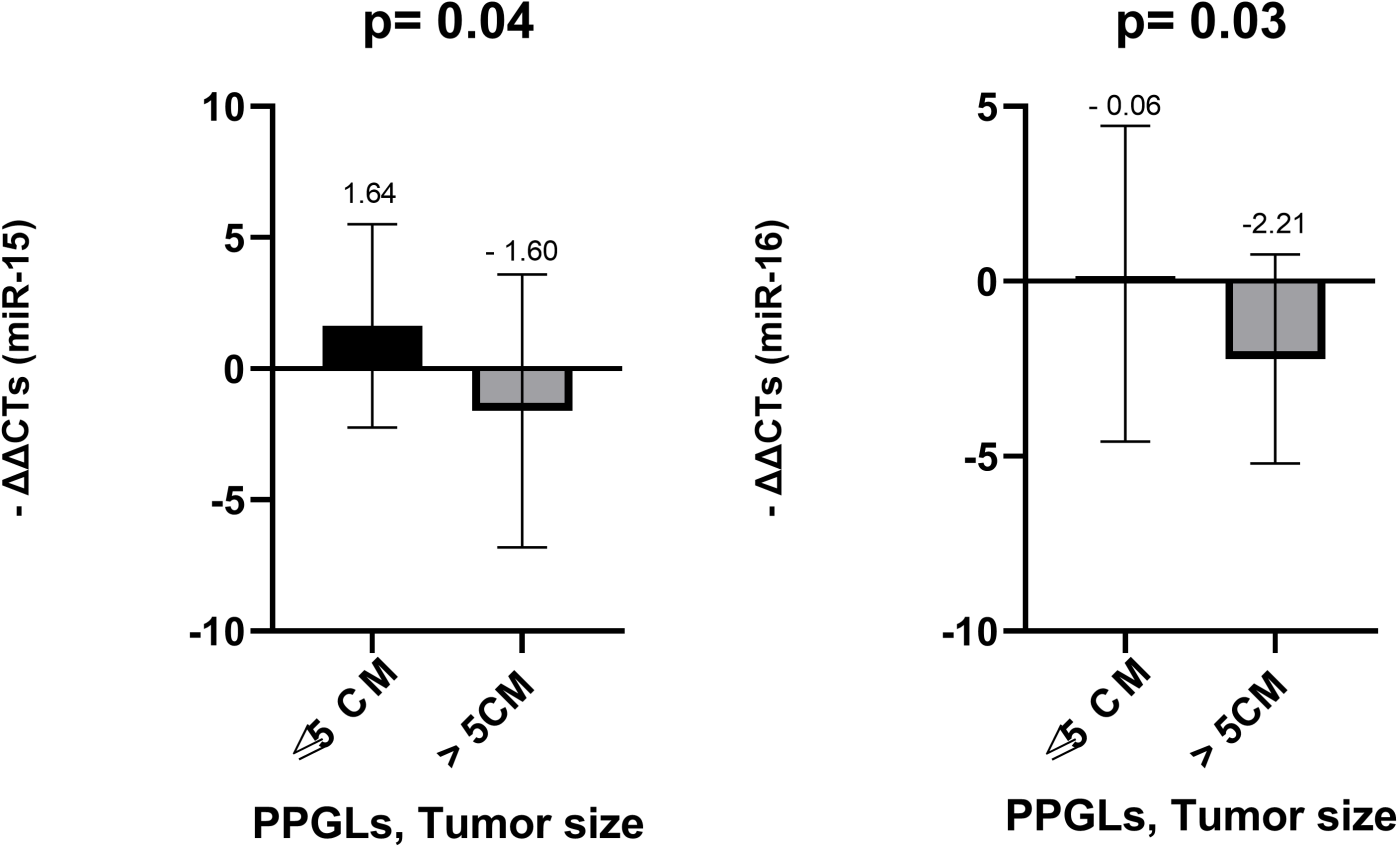
Comparison of miR-15a and miR-16 levels in FFPE among PPGLs with tumor size ≤ 5 and >5cm. *Mann-Whitney test was applied to assess statistical differences between the groups. Error bars represent the standard deviation, and mean values are shown to illustrate the magnitude of each comparison. FFPE, Formalin-Fixed Paraffin-Embedded; PPGLs, Pheochromocytomas and Paragangliomas.

**Figure 7:**
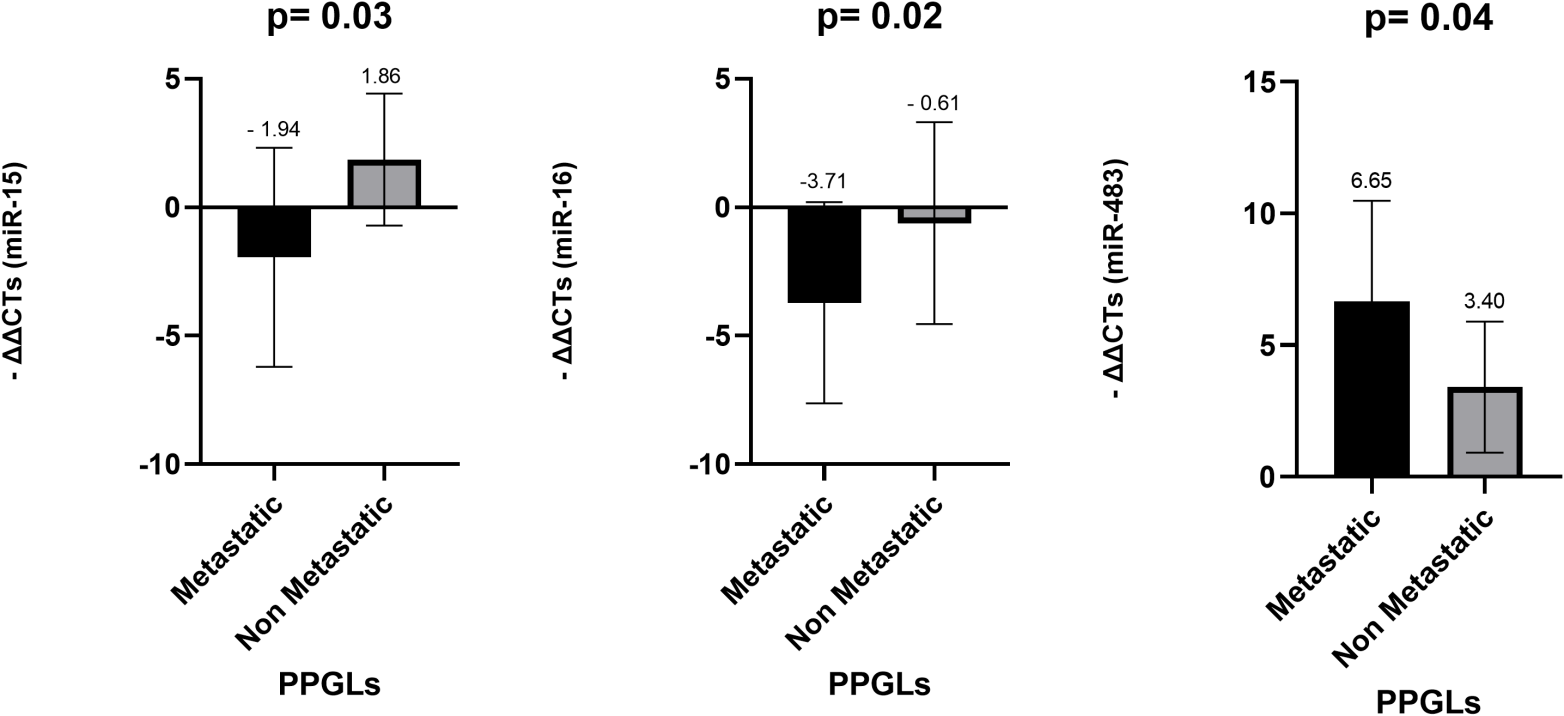
Comparison of miR-15a, miR-16 and miR-483-5p levels in FFPE among metastatic and non-metastatic PPGLs. *Mann-Whitney test was applied to assess statistical differences between the groups. Error bars represent the standard deviation, and mean values are shown to illustrate the magnitude of each comparison. FFPE, Formalin-Fixed Paraffin-Embedded; PPGLs, Pheochromocytomas and Paragangliomas.

We also examined differences in miRNA expression based on tumor size, comparing PPGLs smaller than 5 cm to larger than 5 cm. Among the miRNAs studied, miRNA-15a (p = 0.040) and miRNA-16 (p =0.033) were significantly upregulated in tumors smaller than 5cm (**Figure 6**). miRNA-101 (p = 0.149), miRNA-183 (p = 0.458) and miRNA-483-5p (p = 0.191) did not demonstrate any significant difference (**Figure 7**).

No statistically significant difference of the miRNA expression was found among patients with germline pathogenic mutation and those without an identified known mutation.

#### 3.3.3 miRNA expression and metastatic disease

The comparative analysis of miRNA expression levels between metastatic and non-metastatic PPGL revealed that miR-15a (p = 0.031) and miR-16 (p = 0.027) demonstrated statistically significant overexpression in non-metastatic PPGL compared to metastatic PPGL. miRNA-483-5p presented statistically significant upregulation in metastatic PPGL (p = 0.045), whereas miR-101(p = 0.486) and miR-183 (p = 0.981) exhibited no significant difference between the two groups (**Figure 7**).

**Figure 8:**
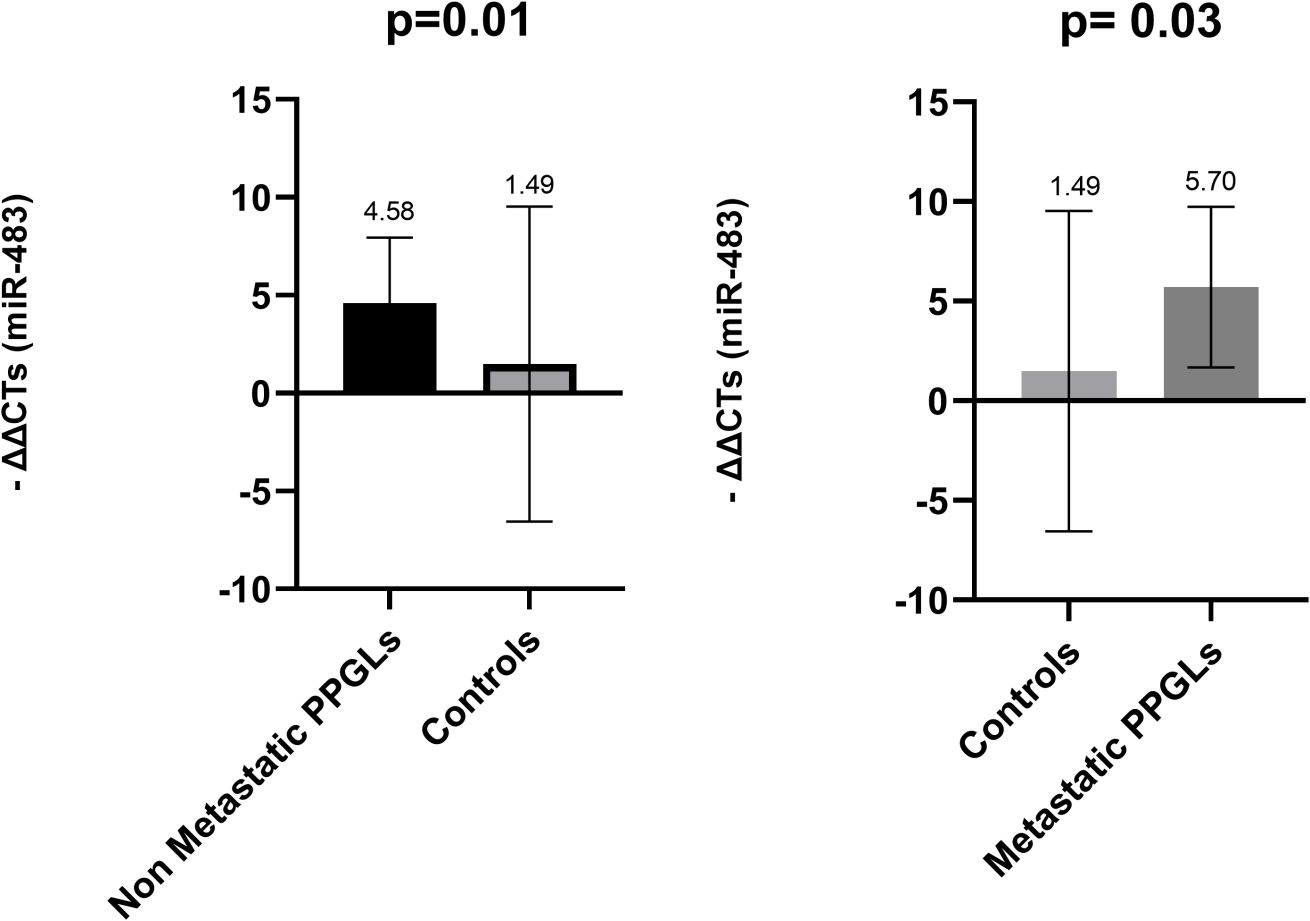
Comparison of miR-483-5p levels in FFPE among metastatic PPGLs, non-metastatic PPGLs and controls. *Kruskal - Wallis test was applied to assess statistical differences between the groups. Error bars represent the standard deviation, and mean values are shown to illustrate the magnitude of each comparison. FFPE, Formalin-Fixed Paraffin-Embedded; PPGLs, Pheochromocytomas and Paragangliomas.

When comparing with normal adrenal medulla, no significant differences were observed for miR-15a (p = 0.485), miR-16 (p = 0.397), miR-101 (p = 0.652), or miR-183 (p = 0.981) in metastatic and non-metastatic PPGLs. However, miR-483-5p was significantly overexpressed in non-metastatic PPGL compared to controls (adjusted p = 0.014) and in metastatic PPGLs compared to controls (adjusted p = 0.030) (**Figure 8**).

All results are summarized in **Table 2**, which provides an overview of the miRNAs that were increased, decreased, or unchanged across the key comparisons. Furthermore, figures illustrating the non-significant comparisons are provided in the **Supplementary Material** (**Supplementary Figures 1–13**).

**Table 2:** Summary of mi RNA expression patterns.

Comparison	miR-15a	miR-16	miR-101	miR-183	miR-483-5p
PPGLs vs normal adrenal medulla	Unchanged	Unchanged	Unchanged	Unchanged	Upregulated
PHEOs vs controls	Unchanged	Unchanged	Unchanged	Upregulated (borderline)	Upregulated
PGLs vs controls	Unchanged	Unchanged	Unchanged	Unchanged	Upregulated
HNPGL vs non-HNPGL	Upregulated in HNPGL	Upregulated in non -HNPGL	Unchanged	Unchanged	Unchanged
Biochemically negative vs positive PPGLs	Unchanged	Unchanged	Upregulated (borderline)	Upregulated	Upregulated
PGLs (GAPP >4 vs ≤4)	Downregulated in GAPP>4	Unchanged	Unchanged	Unchanged	Unchanged
PHEOs (PASS >4 vs ≤4)	Unchanged	Unchanged	Unchanged	Upregulated in PASS>4	Unchanged
PHEOs (PASS >6 vs ≤6)	Unchanged	Unchanged	Upregulated in PASS>6	Upregulated in PASS>6	Unchanged
Ki-67 >4% vs ≤4%	Unchanged	Unchanged	Upregulated	Unchanged	Upregulated
Tumor size <5 cm vs ≥5 cm	Upregulated in tumors < 5 cm	Upregulated in tumors < 5 cm	Unchanged	Unchanged	Unchanged
Germline mutation vs no mutation	Unchanged	Unchanged	Unchanged	Unchanged	Unchanged
Metastatic vs non-metastatic PPGLs	Downregulated in metastatic	Downregulated in metastatic	Unchanged	Unchanged	Upregulated in metastatic
Metastatic PPGLs vs controls	Unchanged	Unchanged	Unchanged	Unchanged	Upregulated
Non-metastatic PPGLs vs controls	Unchanged	Unchanged	Unchanged	Unchanged	Upregulated

Upregulated = increased expression; Downregulated = decreased expression; Unchanged = no statistically significant difference; borderline = trend without statistical significance/p=0.05

PPGLs, Pheochromocytomas and Paragangliomas; PHEOs, Pheochromocytomas; PGLs, Paragangliomas; HNPGLs, head and neck paragangliomas; PASS, Pheochromocytoma of the Adrenal gland Scaled Score; GAPP, Grading system for Adrenal PHEO and PGL; Biochemically positive: plasma or urinary catecholamine/metanephrine levels ≥3–5× upper normal limit (liquid chromatography–tandem mass spectrometry, LC–MS/MS), Biochemically negative: within normal range.

## 4 Discussion

In our study, we demonstrated distinct molecular expression patterns of specific miRNAs in relation to proliferative index, tumor size, metastatic potential, and histological subtype. Data regarding prognostic markers of clinical behavior in PPGLs still remain scarce. Among them, germline mutations in *SDHB* are the most consistently recognized predictor of aggressive or metastatic disease (21–26). Additional factors proposed to influence metastatic potential include non-adrenal tumor location, larger tumor size, biochemical profile (noradrenergic or dopaminergic versus adrenergic), and somatic alterations in genes involved in telomere maintenance, such as ATRX mutations and TERT overexpression (25, 27–35).

In our study, miR-483-5p was significantly overexpressed in PPGL tissues compared to normal adrenal medulla, particularly in biochemically negative tumors as well as in metastatic tumors. miR-15a was significantly overexpressed in HNPGLs whereas miR-16 was overexpressed in non-HNPGLs. Furthermore, miR-183 and miR-101 were significantly upregulated in PHEOs with PASS >6, while miR-15a was downregulated in PGLs with GAPP > 4. Interestingly, miR-15a and miR-16 were significantly overexpressed in non-metastatic compared to metastatic PPGLs.

In the literature, data regarding the association between tumor size and metastatic potential in PPGLs remain conflicting. While some studies (9) suggest that larger tumor size is linked to more aggressive behavior and increased risk of metastasis, others failed to demonstrate a statistically significant correlation (19, 36). Our findings showed that miRNA-15a and miR-16 were upregulated in smaller tumors (<5 cm).

miRNA483-5p increased expression in metastatic PPGLs compared to non-metastatic, was correlated with insulin growth factor 2 (IGF2) amplification, as both genes are co-expressed from the same genomic locus (13). Recently, miR-483-5p was also identified as a marker of worse disease-free survival, reinforcing its potential role as a prognostic biomarker. In another study, circulating miR-483-5p was the best predictor of metastatic potential in PPGLs (AUC-ROC 0.64, 95%CI 0.52-0.77) (37). miR-483-5p overexpression in metastatic PPGLs is in agreement with previous reports and with circulating-miRNA data (38).

Consistent with previous findings, miR-15a and miR-16 also demonstrated a significant downregulation in metastatic versus non-metastatic PPGLs (13). *In vitro* studies indicated that these two particular miRNAs promoted growth inhibition, induced cell cycle arrest and apoptosis through their target cyclin D1 when transfected into the rat PC12 PCC cell line (6). Low miR-15a expression associated with high expression of IGF2 could differentiate metastatic from non-metastatic tumors. However, it is important to note that 20% of metastatic cases were misclassified as low-risk, confirming its existing limitations in its predictive sensitivity. Thus, our observations that miR-15a is downregulated in PGLs with a GAPP score >4 and miR-15a/miR-16 are overexpressed in non-metastatic tumors are consistent with those prior tissue-based reports and supports their putative tumor-suppressor role in PPGL clinical behavior.

In another study, whole-genome microarray profiling revealed significant overexpression of miR-101, miR-183, and miR-483-5p in metastatic PPGLs compared to non-metastatic ones (14). In our cohort we found that miR-183 and miR-101 were upregulated in tumors with higher PASS scores predicting thus a more aggressive histopathologic features and clinical behavior.

Data comparing miRNA expression between HNPGLs and non-HNPGLs are rare. Most studies focus on genotype (e.g., SDHx status) and broader transcriptomic or proteomic differences rather than miRNA expression (39). Because HNPGLs are more frequently associated with SDHx mutations, genotype-related miRNA signatures may confound simple anatomic comparisons. In this respect, our finding of differential miR-15a/miR-16 expression between non-HNPGL (one patient with SDHA mutation, one with SDHB, and two with SDHD) and HNPGL (one patient with SDHD mutation) tumors should be interpreted in the context of the genotypes in each group. However, the small number of patients doesn’t allow any further statistical analysis and robust conclusions.

miRNA analyses stratified by biochemical phenotype (adrenergic vs noradrenergic vs dopaminergic vs biochemically silent) are sparse. A few circulating miRNA studies have suggested possible associations with malignant behavior but do not provide robust, replicated comparisons across biochemical subtypes (40). Therefore, while our observation of upregulation of miR-483-5p and miR-183 in biochemically-negative tumors is intriguing, published evidence directly comparing miRNA patterns by catecholamine phenotype is limited; this is an important gap that larger, genotype- and stage-adjusted studies should address.

Regarding immunohistochemical markers, a recent study involving 100 PPGL patients (10% metastatic) revealed that PD-L1 expression was present in 18% of tumors and appeared to be independent of traditional adverse pathological features or hypoxia biomarkers. In contrast, PD-L2 expression in PPGL tumors was significantly associated with capsular and vascular invasion, necrosis, malignant behavior, and increased expression of hypoxia-related markers. Importantly, PD-L2 expression was also predictive of significantly shorter survival. These data suggest that there is an immunobiological heterogeneity among PPGL tumors allowing the consideration of immune-checkpoint targeting in selected patients (15). In our study, PDL-1 expression was detected in approximately 19% of the tested PPGL tissue samples. Most genomic analyses have also shown low tumor mutational burden (TMB) and microsatellite instability (MSI) in PPGLs, suggesting that MSI-high disease is uncommon and that classical MSI-driven sensitivity to immune checkpoint blockade is unlikely to be a major target for most PPGLs (41). In our cases, MSI was not detected in any of the PPGL tissues in our study, as assessed by immunohistochemistry for mismatch repair protein expression (MLH1, PMS2, MSH2, MSH6).

Although our study provides preliminary insights into the use of miRNA expression in tissue levels in patients with PPGLs as a non-invasive technique to predict the clinical behavior of these tumors we acknowledge certain limitations. Indeed, the limited availability of tissue samples—due to insufficient specimens or storage issues— along with technical difficulties in miRNA isolation, reduced the accuracy of our statistical analyses. Furthermore, different tissue block storage conditions as well as period of storage between control and PPGLs samples could be a potential limitation in the interpretation of miRNA expression. Our study is limited by the lack of synchronous circulating-miRNA measurements (blood) and by sample-size constraints for certain subgroup analyses (biochemical subtypes, PASS/GAPP subgroups, and HNPGL vs non-HNPGL). In addition, comparisons between circulating and tissue miRNA levels should be undertaken with caution because tissue and plasma miRNA expression often differ and, in some tumor types, are inversely correlated. Finally, genotype (e.g., SDHx status) which may confound miRNA associations, was not available in all PPGL tissues, limiting further statistical analyses and robust conclusions.

## 5 Conclusions and future directions

Taken together, our tissue miRNA results largely align with prior reports that implicate miR-483-5p, miR-101, miR-183, miR-182, and the miR-15a/16 cluster in PPGL aggressiveness and metastasis. The evidence is strongest for tissue-based miR-483-5p (upregulated) and miR-15a/16 (downregulated) expression in metastatic tumors compared to non-metastatic ones. Furthermore, we have shown that the expression of miR-483-5p and miR-183 is associated with the biochemical profile of PPGL tumors, which represents a novel finding given the scarcity of data currently available in the literature. No significant differences in the expression of the miRNAs analyzed were found for genotype and anatomical site (HNPGL vs non-HNPGL). Future work should integrate tissue and plasma miRNA profiling with genotype, PASS/GAPP scores, and standardized PD-L1/PD-L2 and MSI/TMB assessments in larger prospective series to validate the prognostic value of these markers and to evaluate their clinical utility.

## Data Availability

The original contributions presented in the study are included in the article/**Supplementary Material**. Further inquiries can be directed to the corresponding author/s.
