# Supplementary material for: Clinicopathological data and the role of miRNA expression in patients with pheochromocytomas/paragangliomas
Source: Front Endocrinol (Lausanne). 2025 Dec 4;16:1716806. doi: 10.3389/fendo.2025.1716806 (PMC12711539; doi:10.3389/fendo.2025.1716806)
Supplement: Supplementary file 1 [file DataSheet1.docx]

Supplementary Material

**SUPPLEMENTARY TABLES**

**Supplementary Table 1. Clinical and histopathological Characteristics of PPGL Patients (n = 130)**

| **Patient Characteristics** | **Value** |
| --- | --- |
| Age, median (range: min-max), years | 48 (15–78) |
| Sex, (m: f) n (%) | 54/130 (41.5 %) : 76/130 (58.5 %) |
| Tumor type, n (%) |  |
| — PGLs | 76/130 (58.5 %) |
| — Unilateral PHEOs | 49/130 (37.7 %) |
| — Bilateral PHEOs | 4/130 (3 %) |
| — Both PGL and PHEO | 1/130 (0.8 %) |
| Symptomatic at diagnosis, n (%) | 70/130 (53.8 %) |
| Biochemical testing, n (%) | 102/130 (78.5 %) |
| -Positive, n (%) | 59/102 (57.8 %) |
| PHEOs : PGLs | 37/59: 22 /59 |
| **Tumor and Tissue Characteristics**  Primary tumor size, cm, median (range: min-max)  Ki-67 index %, median (range: min-max)  PASS score (PHEOs), median (range: min-max)  GAPP score (PHEOs-sympathetic PGLs), median (range: min-max)  **Treatment Type**  Surgery*, n (%)  — PHEOs (operated / total)  — PGLs (operated / total)  Overall survival, n (%)  Follow-up, median in months (range: min-max) | 5.4 (1–15)  3 (1–20)  6 (1–12)  4 (2–8)  95/130 (73 %)  52/95 (54.7 %)  43/95 (45.3 %)  125/130 (96.1 %)  45 (12-260) |

*Surgical intervention includes adrenalectomy and/or extra-adrenal tumor excision. Abbreviations: PPGL = Pheochromocytoma and Paraganglioma, PHEOs = Pheochromocytomas PGLs = Paragangliomas, PASS = Pheochromocytoma of the Adrenal gland Scaled Score, GAPP = Grading system for Adrenal Pheochromocytoma and Paraganglioma

**Supplementary Table 2. Metastatic Disease and Survival Outcomes (n = 28/130 patients)**

| **Variable** | **Value** |
| --- | --- |
| Metastatic disease, n (%) | 28/130 (21.5 %) |
| — Synchronous metastases, n (%) | 6/28 (21.4 %) |
| — New metastases during follow-up, n (%)  **Management of Metastatic Disease**  **Local Treatments**  Radiotherapy  Second surgery  Third surgery  **Systemic Treatments**  Chemotherapy (CVD / Temozolomide)  PRRTs / ^131^I-MIBG  MTT (Sunitinib / Everolimus) | 22/28 (78.6%)  7/28 (25 %)  10/28 (35.7 %)  3/28 (10.7 %)  **n (%) 1st Line 2nd Line 3rd Line**  10/28 (35.7 %) 7 2 1  10/28 (35.7 %) 7 1 2  5/28 (17.9 %) 2 1 2 |
| Overall Median PFS in months (range: min-max) | 70.8 (5.8- 182.4) |
| Median follow-up in months (range: min-max) | 84 (12- 216) |
| **Progression-Free Survival by Treatment Type**  Chemotherapy (CVD)  PRRT  ^131^I-MIBG  MTT | **Median PFS (months) Range: min-max**  5.39 3–55  7.9 6.6–180  36.6 16–124  6.96 2.7–55.6 |

The overall median progression-free survival (PFS) of the subgroup of patients with metastatic disease was 70.8 months during a median follow-up time of 84 months. The median PFS varied according to treatment modality; for patients treated with chemotherapy was 5.39 months, for those treated with PRRTs was 7.9 months, with 131I-MIBG was 36.6 months and with MTT was 6.96 months.

Abbreviations: PFS = Progression-Free Survival, CVD = Cyclophosphamide – Vincristine - Dacarbazine, PRRT = Peptide Receptor Radionuclide Therapy; ^131I-MIBG = Iodine-131-metaiodobenzylguanidine; MTT = Molecular Targeted Therapy

**Supplementary Table 3. Genetic Findings**

| **Genetic Analysis** | **Value** |
| --- | --- |
| Patients tested genetically, n (%) | 61/130 (46.9 %) |
| Mutation-positive patients, n (%) | 35/61 (57.4 %) |
| Gene mutations detected, n |  |
| — SDHA | 2 |
| — SDHB | 7 |
| — SDHC | 1 |
| — SDHD | 14 |
| — RET | 5 |
| — NF1 | 4 |
| — VHL | 1 |
| — EPAS1 | 1 |

Abbreviations: SDHx = Succinate Dehydrogenase subunit genes, RET = Rearranged during Transfection protooncogene, NF1 = Neurofibromatosis type 1, VHL=von Hippel-Lindau, EPAS1 = Endothelial PAS Domain-Containing Protein 1

**SUPPLEMENTARY FIGURES**

**
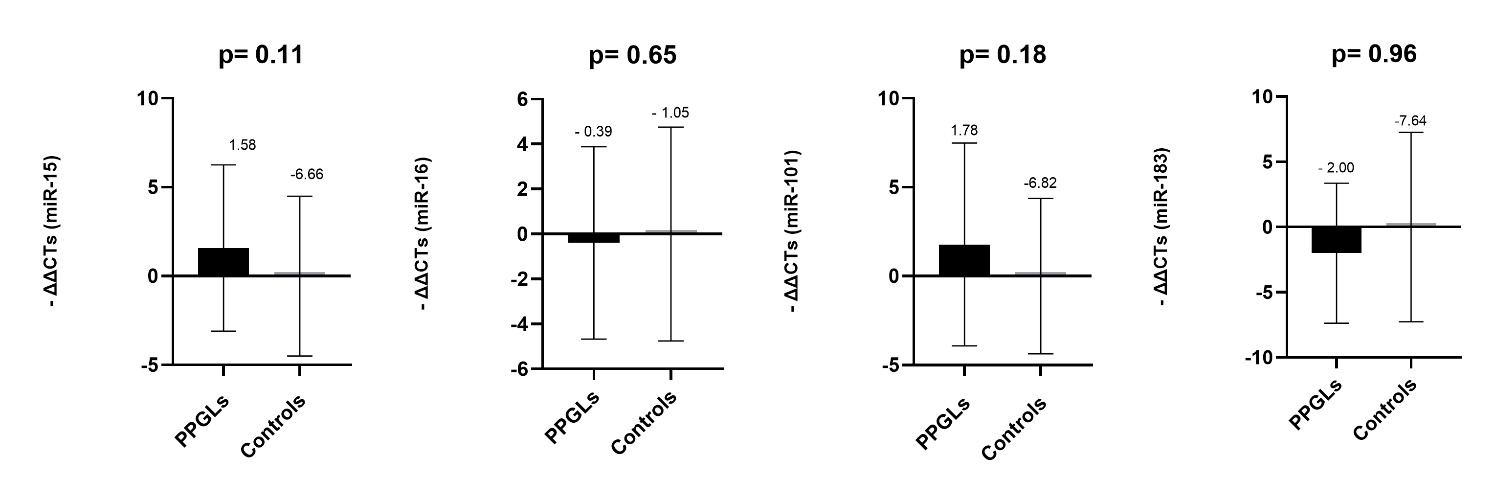
**

**SUPPLEMENTARY FIGURE 1.**

Comparison of miR-15a (A), miR-16 (B), miR-101 (C) and miR-183 (D) levels in FFPE among PPGLs and Controls. *Kruskal - Wallis test was applied to assess statistical differences between the groups. Error bars represent the standard deviation, and mean values are shown to illustrate the magnitude of each comparison. Abbreviations: FFPE: Formalin-Fixed Paraffin-Embedded, PPGLs: Pheochromocytomas and Paragangliomas.

**
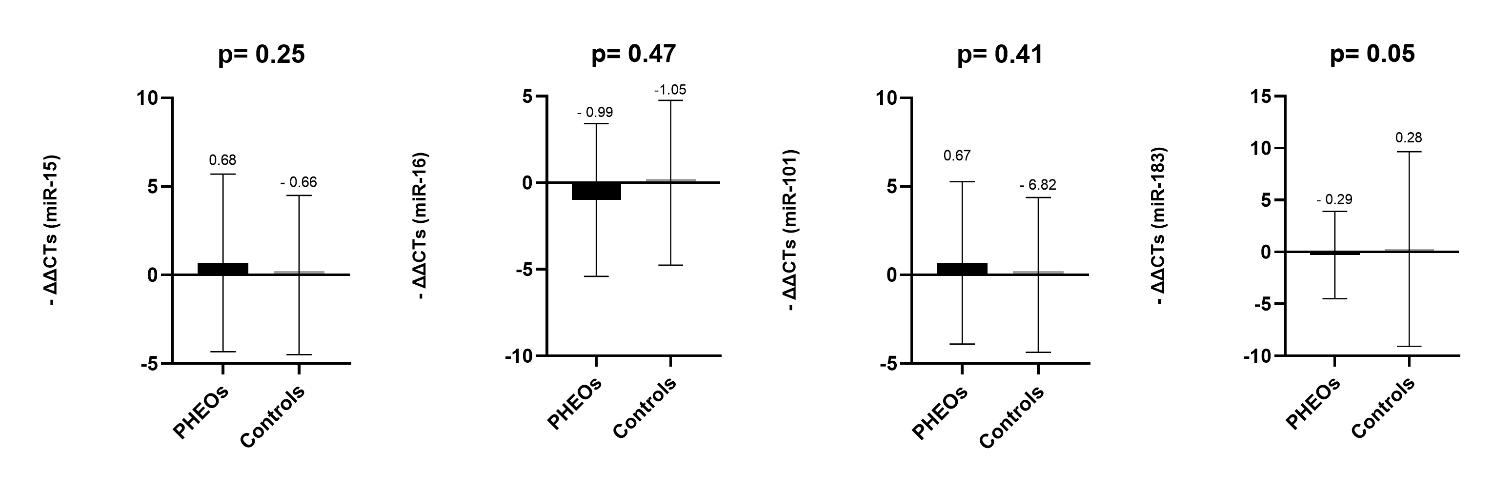
**

**SUPPLEMENTARY FIGURE 2.**

Comparison of miR-15a (A), miR-16 (B), miR-101 (C) and miR-183 (D) levels in FFPE among PHEOs and Controls. *Kruskal - Wallis test was applied to assess statistical differences between the groups. Error bars represent the standard deviation, and mean values are shown to illustrate the magnitude of each comparison. Abbreviations: FFPE: Formalin-Fixed Paraffin-Embedded, PHEOs: Pheochromocytomas

**
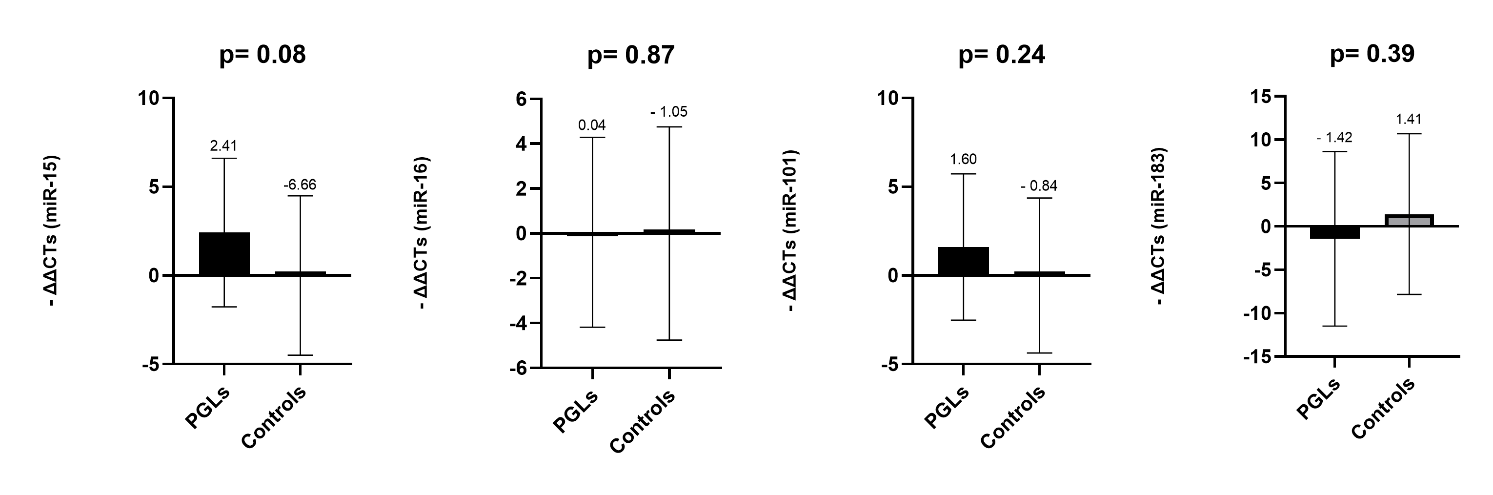
**

**SUPPLEMENTARY FIGURE 3.**

Comparison of miR-15a (A), miR-16 (B), miR-101 (C) and miR-183 (D) levels in FFPE among PGLs and Controls. *Kruskal - Wallis test was applied to assess statistical differences between the groups. Error bars represent the standard deviation, and mean values are shown to illustrate the magnitude of each comparison. Abbreviations: FFPE: Formalin-Fixed Paraffin-Embedded, PGLs: Paragangliomas.

**
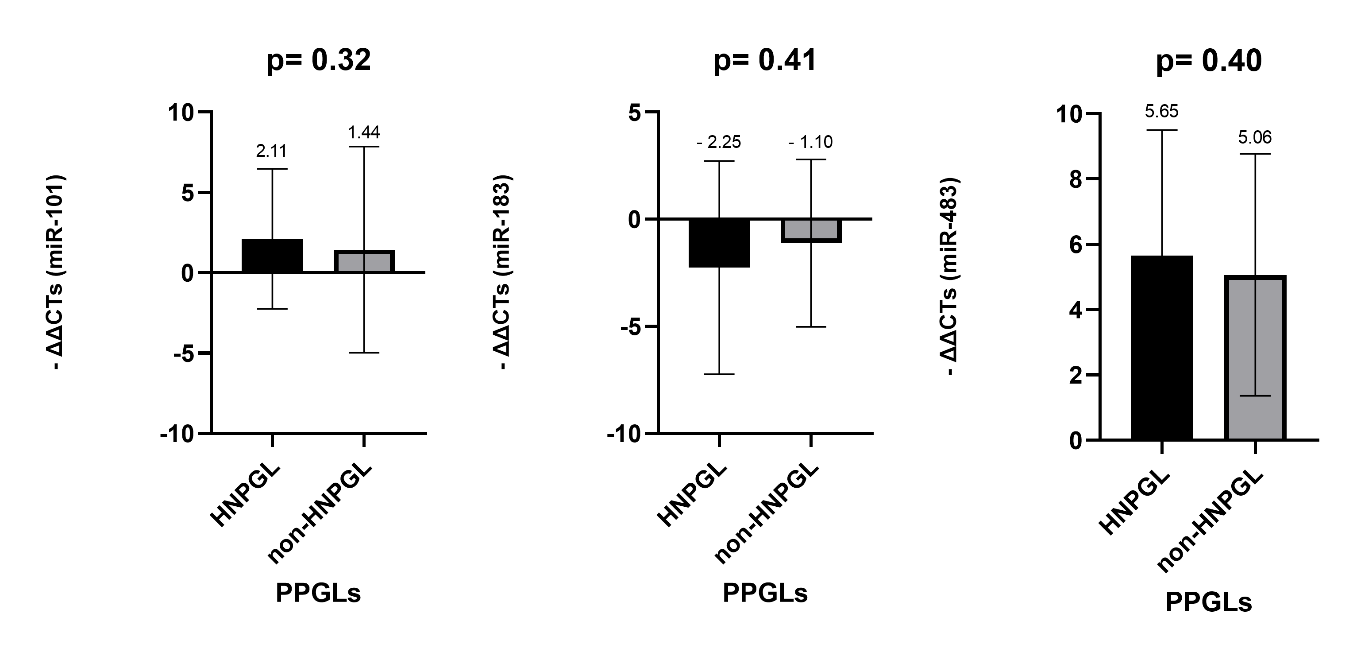
**

**SUPPLEMENTARY FIGURE 4.**

Comparison of miR-101 (A), miR-183 (B) and miR-483-5p (C) levels in FFPE among HNPGLs and non-HNPGLs. *Mann-Whitney test was applied to assess statistical differences between the groups. Error bars represent the standard deviation, and mean values are shown to illustrate the magnitude of each comparison. Abbreviations: FFPE: Formalin-Fixed Paraffin-Embedded, HNPGL: Head and Neck Paragangliomas, PPGLs: Pheochromocytomas and Paragangliomas.


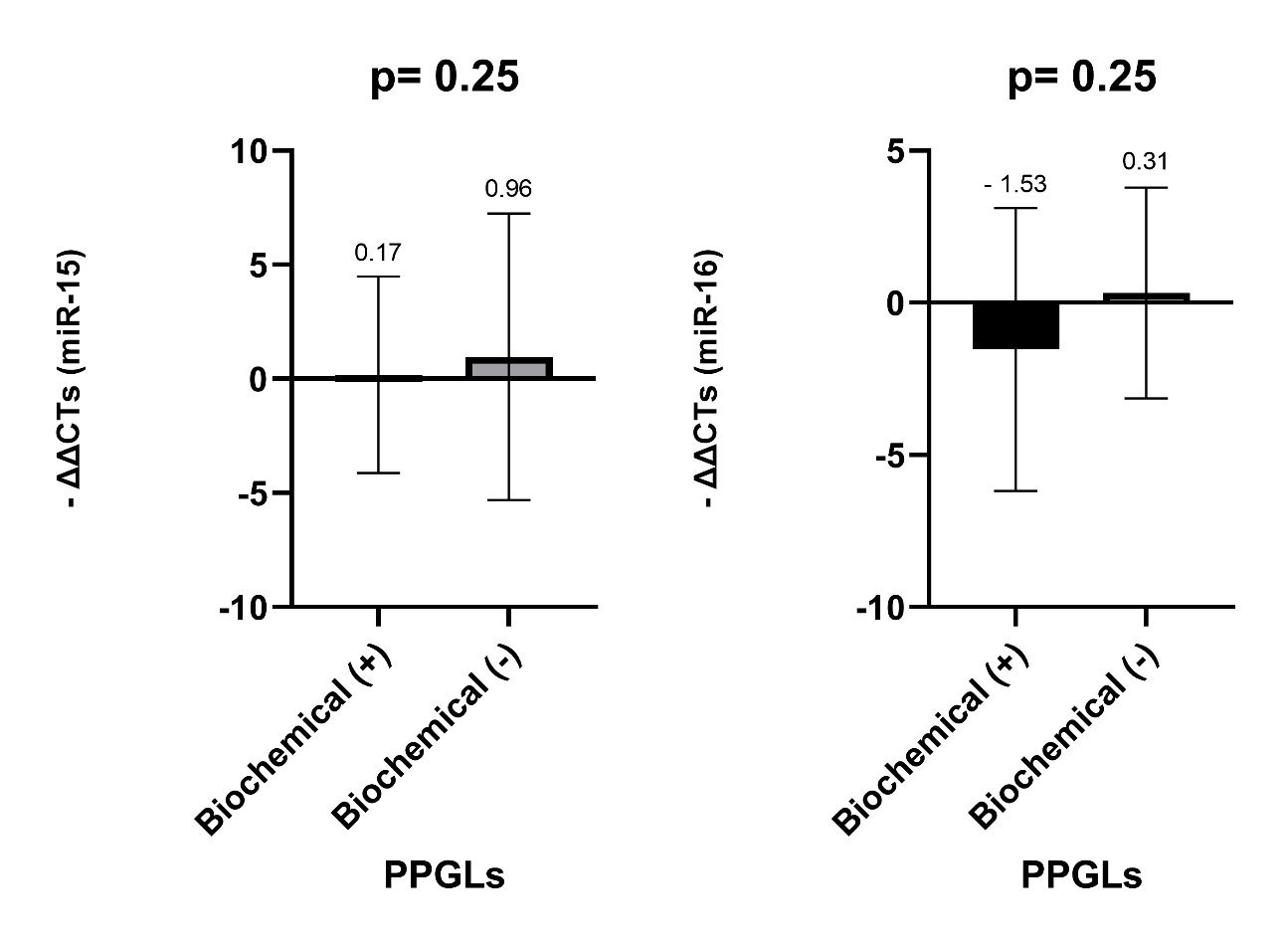


**SUPPLEMENTARY FIGURE 5**.

Comparison of miR-15a (A) and miR-16 (B) levels in FFPE tissues samples among biochemical positive and biochemical negative PPGLs. *Mann-Whitney test was applied to assess statistical differences between the groups. Error bars represent the standard deviation, and mean values are shown to illustrate the magnitude of each comparison Abbreviations: PPGLs: Pheochromocytomas and Paragangliomas, FFPE: Formalin-Fixed Paraffin-Embedded, Biochemical (+): tumors with plasma or urinary catecholamine / metanephrine / normetanephrine levels ≥ 3–5× upper normal limits (LC–MS/MS), Biochemical (-): tumors with normal plasma or urinary catecholamine / metanephrine / normetanephrine levels (LC–MS/MS).


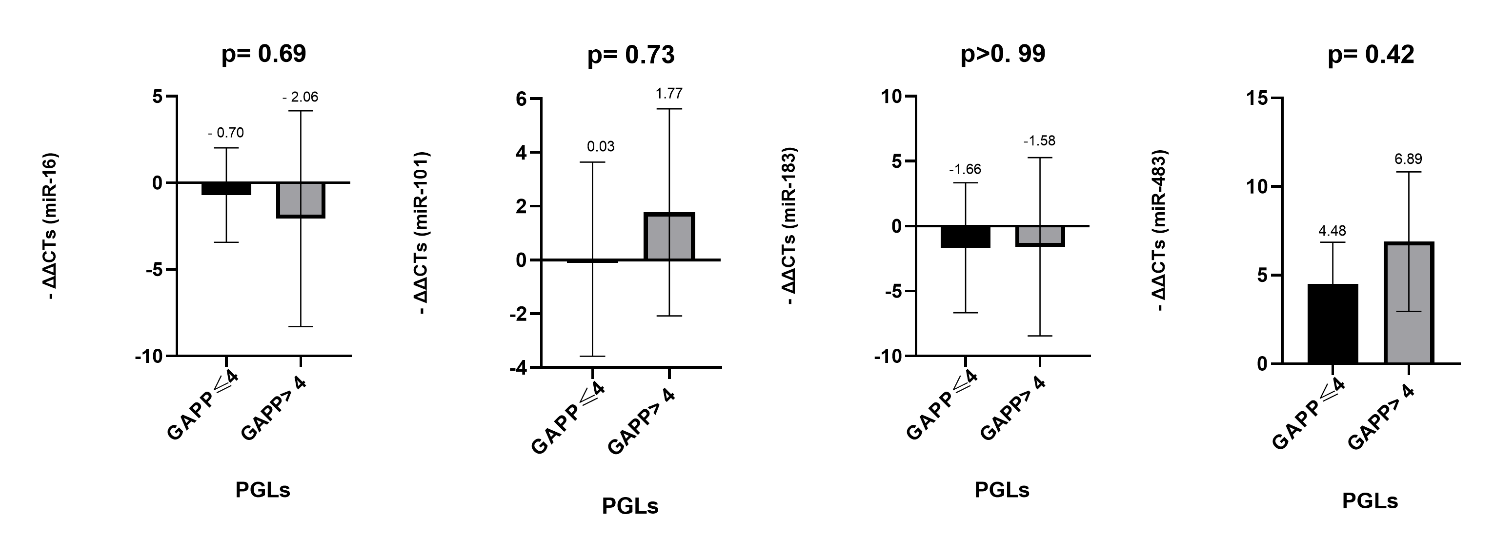


**SUPPLEMENTARY FIGURE 6.**

Comparison of miR-16 (A), miR-101 (B), miR-183 (C) and miR-483-5p (D) levels in FFPE tumors samples among PGLs with GAPP ≤ 4 and GAPP>4. *Mann- Whitney test was applied to assess statistical differences between the groups. Error bars represent the standard deviation, and mean values are shown to illustrate the magnitude of each comparison Abbreviations: FFPE: Formalin-Fixed Paraffin-Embedded, PGLs: Paragangliomas, GAPP: Grading system for Adrenal PHEO and PGL


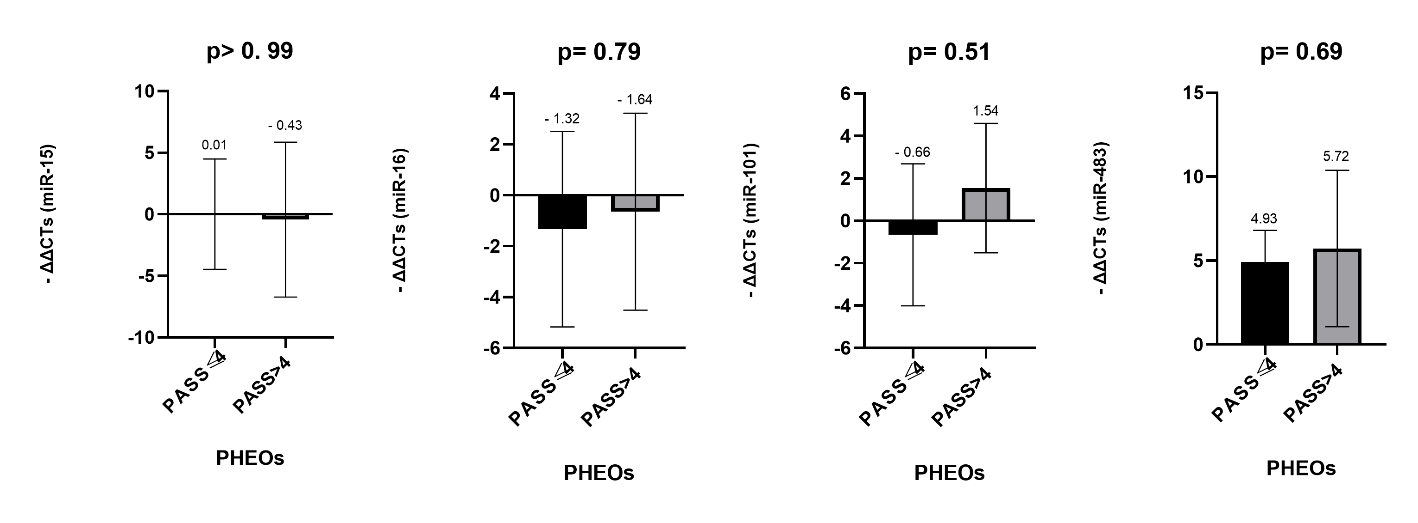


**SUPPLEMENTARY FIGURE 7.**

Comparison of miR-15a (A), miR-16 (B), miR-101 (C) and miR-483-5p (D) levels in FFPE among PHEOs with PASS ≤ 4 and PASS>4.

*Mann-Whitney test was applied to assess statistical differences between the groups. Error bars represent the standard deviation, and mean values are shown to illustrate the magnitude of each comparison. Abbreviations: FFPE: Formalin-Fixed Paraffin-Embedded, PHEOs: Pheochromocytomas, PASS = Pheochromocytoma of the Adrenal gland Scaled Score.


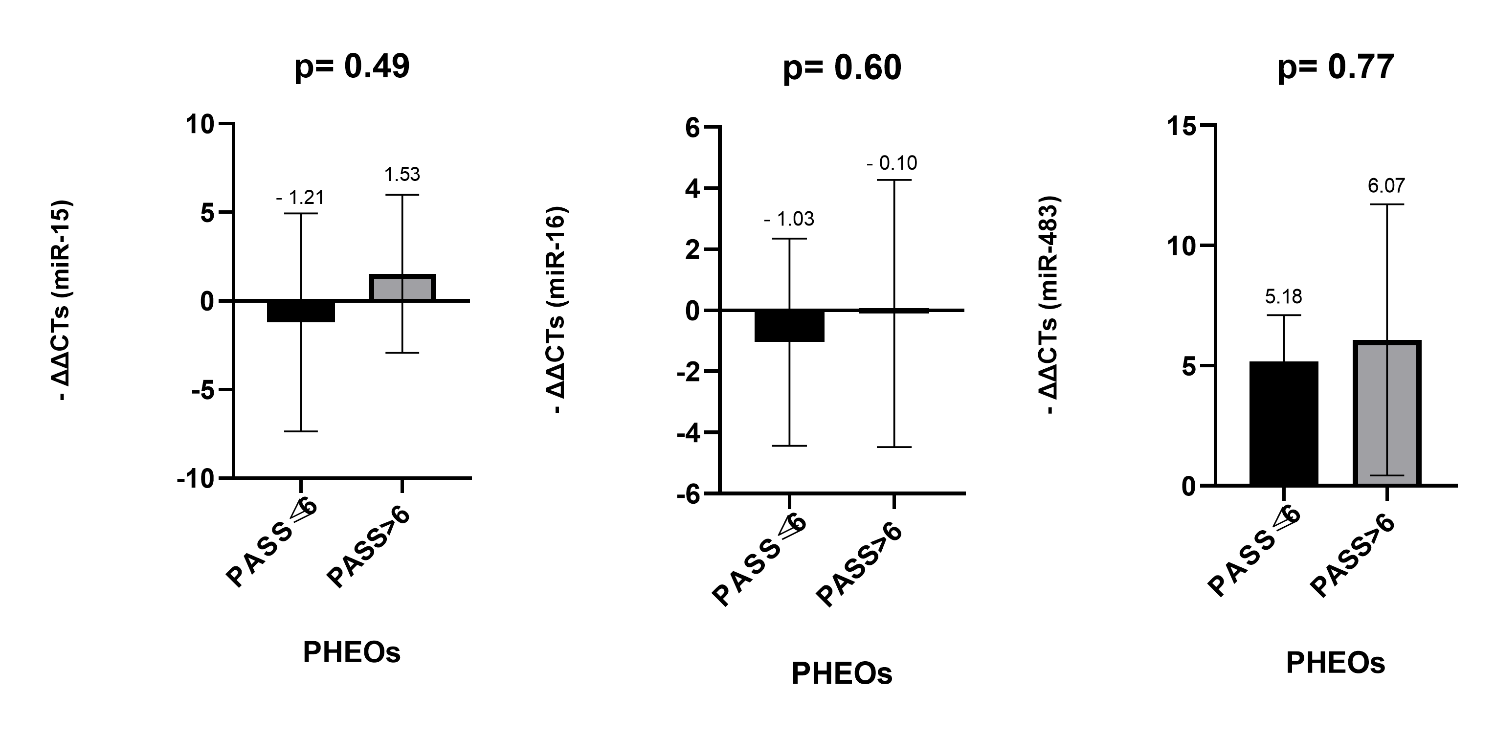


**SUPPLEMENTARY FIGURE 8.**

Comparison of miR-15a (A), miR-16 (B) and miR-483-5p (C) levels in FFPE among PHEOs with PASS ≤ 6 and PASS>6. *Mann-Whitney test was applied to assess statistical differences between the groups. Error bars represent the standard deviation, and mean values are shown to illustrate the magnitude of each comparison. Abbreviations: FFPE: Formalin-Fixed Paraffin-Embedded, PHEOs: Pheochromocytomas, PASS = Pheochromocytoma of the Adrenal gland Scaled Score.

**
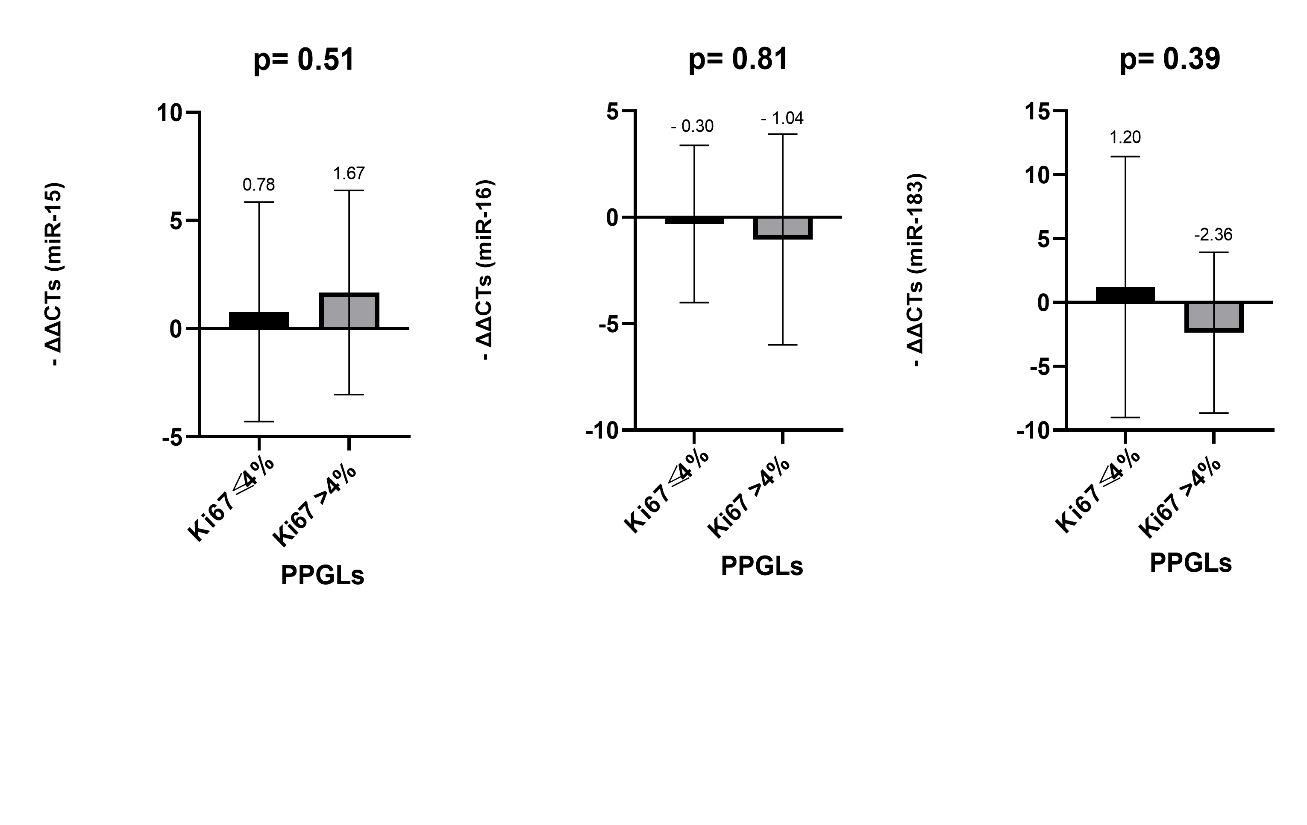
**

**SUPPLEMENTARY FIGURE 9.**

Comparison of miR-15a (A), miR-16 (B) and miR-183 (C) levels in FFPE among PPGLs with Ki67 index ≤ 4% and Ki67 index >4%. *Mann-Whitney test was applied to assess statistical differences between the groups. Error bars represent the standard deviation, and mean values are shown to illustrate the magnitude of each comparison Abbreviations:FFPE: Formalin-Fixed Paraffin-Embedded, PPGLs: Pheochromocytomas and Paragangliomas.


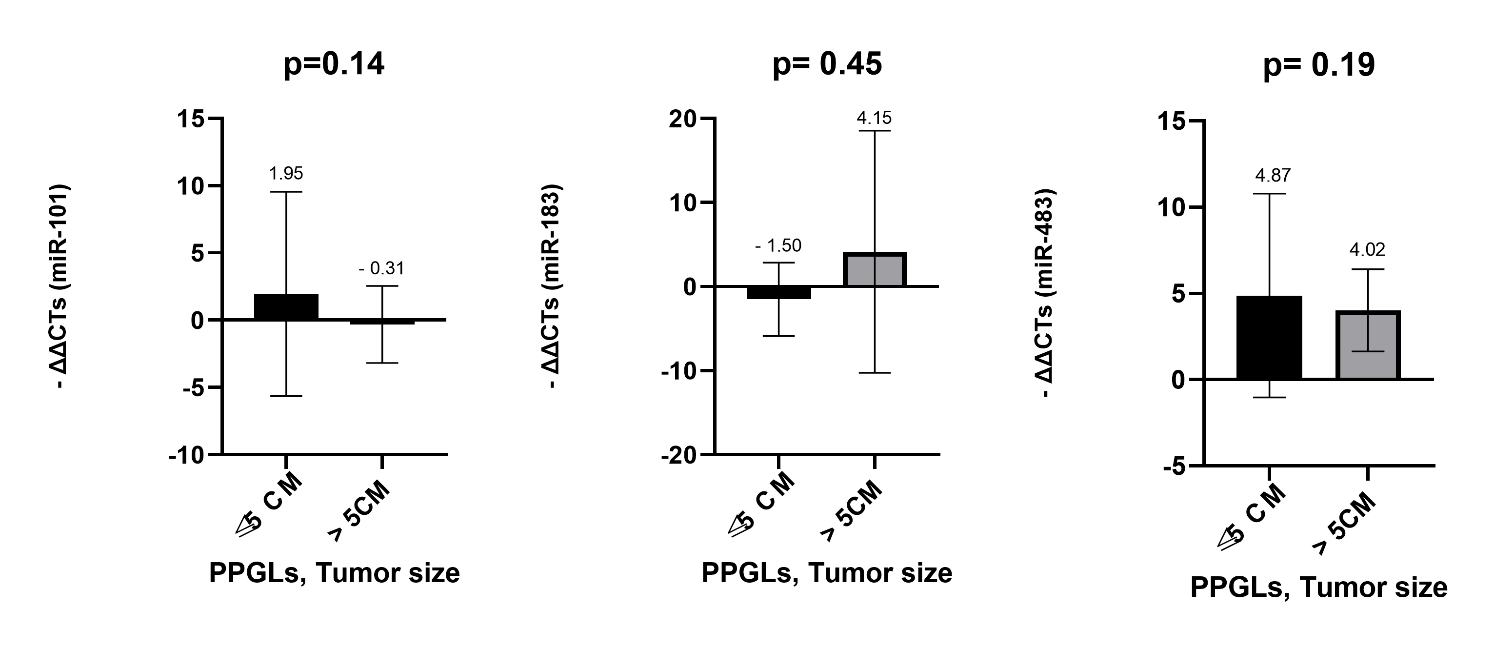


**SUPPLEMENTARY FIGURE 10.**

Comparison of miR-101 (A), miR-183 (B) and miR-483-5p (C) levels in FFPE among PPGLs with tumor size ≤ 5 and >5cm. *Mann-Whitney test was applied to assess statistical differences between the groups. Error bars represent the standard deviation, and mean values are shown to illustrate the magnitude of each comparison. Abbreviations: FFPE: Formalin-Fixed Paraffin-Embedded, PPGLs: Pheochromocytomas and Paragangliomas.


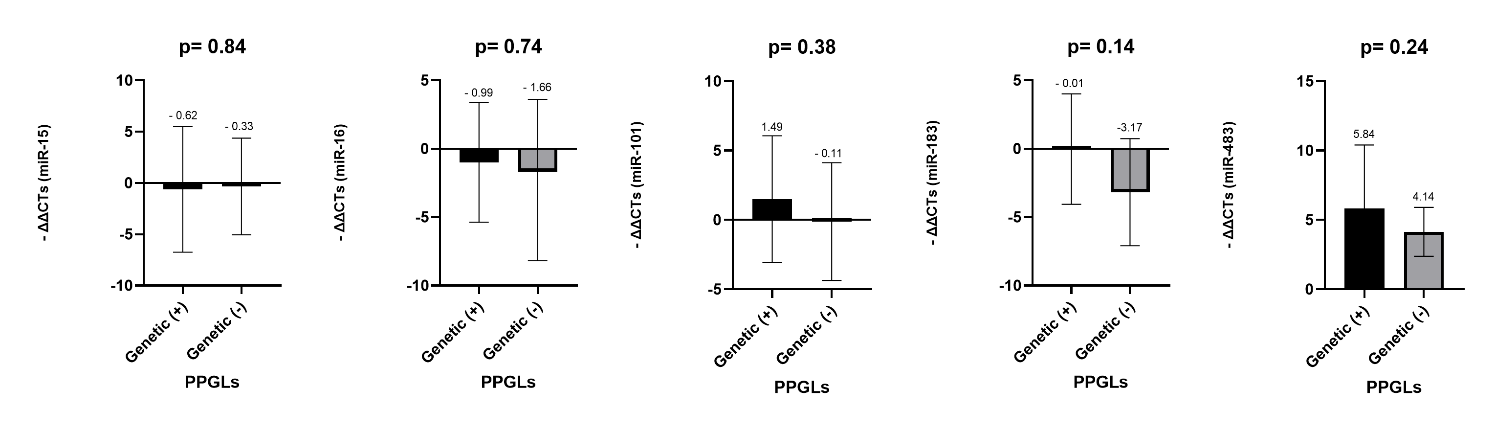


**SUPPLEMENTARY FIGURE 11.**

Comparison of miR-15a (A), miR-16 (B), miR-101 (C), miR-183 (D) and miR-483-5p (E) levels in FFPE among patients with germline pathogenic mutation and those without an identified known mutation. *Kruskal - Wallis test was applied to assess statistical differences between the groups. Error bars represent the standard deviation, and mean values are shown to illustrate the magnitude of each comparison. Abbreviations: FFPE: Formalin-Fixed Paraffin-Embedded, PPGLs: Pheochromocytomas and Paragangliomas, Genetic (+): patients harboring a germline pathogenic mutation, Genetic (-):patients without an identified germline mutation.

**
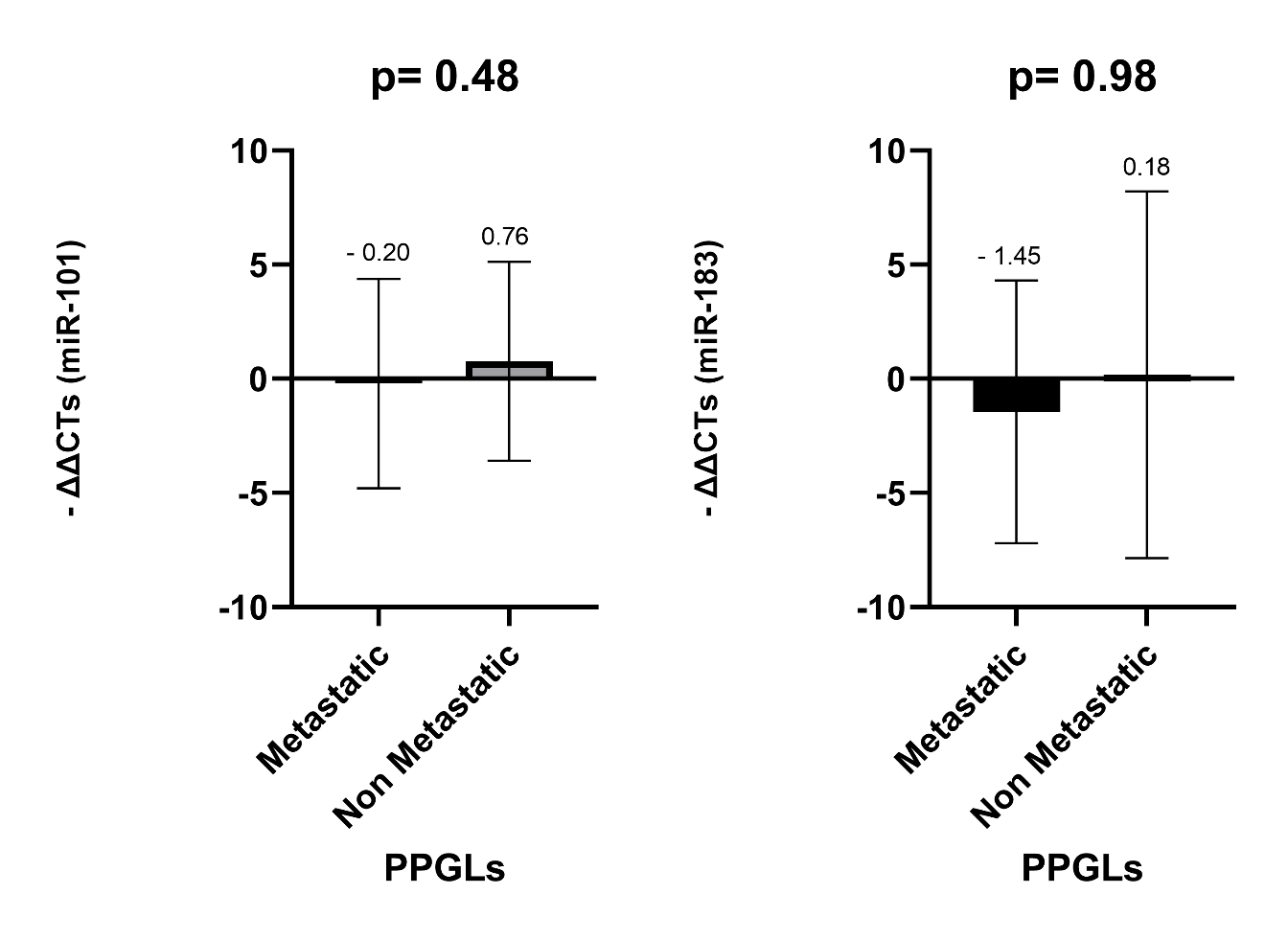
**

**SUPPLEMENTARY FIGURE 12.**

Comparison of miR-101 (A) and miR-183 (B) levels in FFPE among metastatic and non-metastatic PPGLs. *Mann-Whitney test was applied to assess statistical differences between the groups. Error bars represent the standard deviation, and mean values are shown to illustrate the magnitude of each comparison. Abbreviations: FFPE: Formalin-Fixed Paraffin-Embedded, PPGLs: Pheochromocytomas and Paragangliomas.


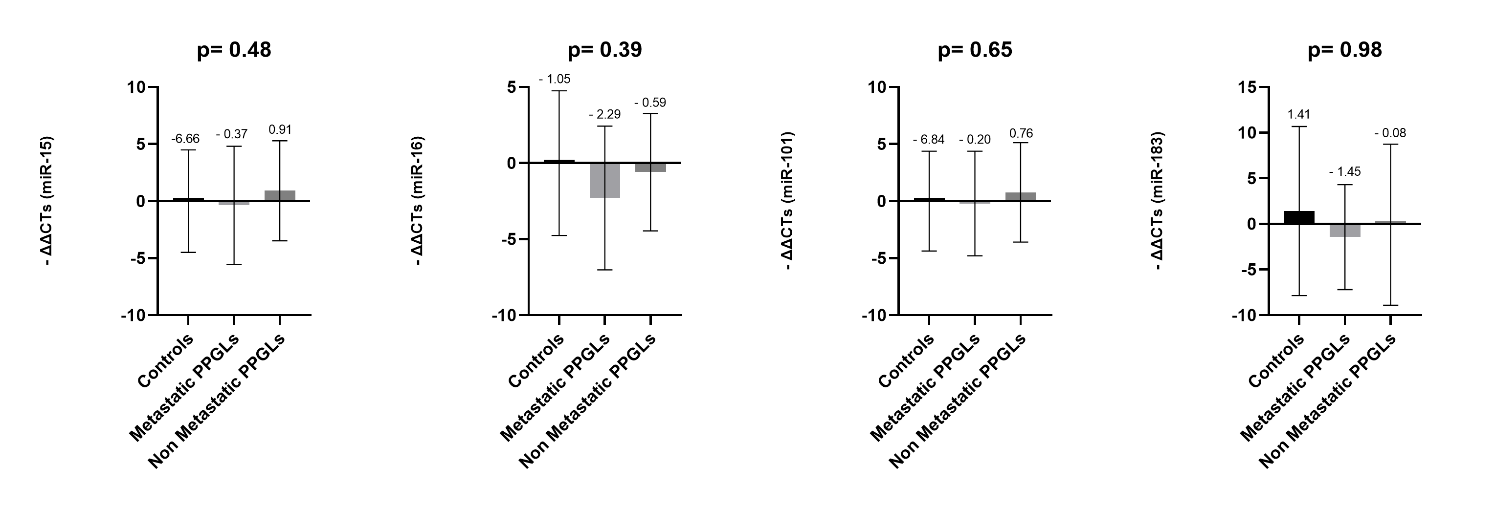


**SUPPLEMENTARY FIGURE 13.**

Comparison of miR-15a (A), miR-16 (B), miR-101 (C) and miR-183 (D) levels in FFPE among metastatic PPGLs, non-metastatic PPGLs and controls. *Kruskal - Wallis test was applied to assess statistical differences between the groups. Error bars represent the standard deviation, and mean values are shown to illustrate the magnitude of each comparison. Abbreviations: FFPE: Formalin-Fixed Paraffin-Embedded, PPGLs: Pheochromocytomas and Paragangliomas.
